# Computational prediction and characterization of cell-type-specific and shared binding sites

**DOI:** 10.1093/bioinformatics/btac798

**Published:** 2022-12-09

**Authors:** Qinhu Zhang, Pengrui Teng, Siguo Wang, Ying He, Zhen Cui, Zhenghao Guo, Yixin Liu, Changan Yuan, Qi Liu, De-Shuang Huang

**Affiliations:** Translational Medical Center for Stem Cell Therapy and Institute for Regenerative Medicine, Shanghai East Hospital, Bioinformatics Department, School of Life Sciences and Technology, Tongji University, Shanghai 200092, China; School of Information and Control Engineering, China University of Mining and Technology, Xuzhou 221116, China; Institute of Machine Learning and Systems Biology, School of Electronics and Information Engineering, Tongji University, Shanghai 201804, China; Institute of Machine Learning and Systems Biology, School of Electronics and Information Engineering, Tongji University, Shanghai 201804, China; Institute of Machine Learning and Systems Biology, School of Electronics and Information Engineering, Tongji University, Shanghai 201804, China; Institute of Machine Learning and Systems Biology, School of Electronics and Information Engineering, Tongji University, Shanghai 201804, China; School of Health Science and Engineering, University of Shanghai for Science and Technology, Shanghai 200093, China; Big Data and Intelligent Computing Research Center, Guangxi Academy of Science, Nanning 530007, China; Translational Medical Center for Stem Cell Therapy and Institute for Regenerative Medicine, Shanghai East Hospital, Bioinformatics Department, School of Life Sciences and Technology, Tongji University, Shanghai 200092, China; EIT Institute for Advanced Study, Ningbo, Zhejiang 315201, China

## Abstract

**Motivation:**

Cell-type-specific gene expression is maintained in large part by transcription factors (TFs) selectively binding to distinct sets of sites in different cell types. Recent research works have provided evidence that such cell-type-specific binding is determined by TF’s intrinsic sequence preferences, cooperative interactions with co-factors, cell-type-specific chromatin landscapes and 3D chromatin interactions. However, computational prediction and characterization of cell-type-specific and shared binding sites is rarely studied.

**Results:**

In this article, we propose two computational approaches for predicting and characterizing cell-type-specific and shared binding sites by integrating multiple types of features, in which one is based on XGBoost and another is based on convolutional neural network (CNN). To validate the performance of our proposed approaches, ChIP-seq datasets of 10 binding factors were collected from the GM12878 (lymphoblastoid) and K562 (erythroleukemic) human hematopoietic cell lines, each of which was further categorized into cell-type-specific (GM12878- and K562-specific) and shared binding sites. Then, multiple types of features for these binding sites were integrated to train the XGBoost- and CNN-based models. Experimental results show that our proposed approaches significantly outperform other competing methods on three classification tasks. Moreover, we identified independent feature contributions for cell-type-specific and shared sites through SHAP values and explored the ability of the CNN-based model to predict cell-type-specific and shared binding sites by excluding or including DNase signals. Furthermore, we investigated the generalization ability of our proposed approaches to different binding factors in the same cellular environment.

**Availability and implementation:**

The source code is available at: https://github.com/turningpoint1988/CSSBS.

**Supplementary information:**

Supplementary data are available at *Bioinformatics* online.

## 1 Introduction

Transcription factors (TFs) play an integral role in the transcriptional regulatory networks by binding to their cognate motifs at specific locations to promote or repress the activities of other TFs, co-factors, chromatin modifiers and the transcriptional machinery (Wang *et al.*, 2012). However, the limited number of TFs encoded by the human genome [∼1600 (Lambert *et al.*, 2018)] is engaged in regulating the transcription activities of a large diversity of cell types, resulting in each individual TF being often reused in multiple distinct cell types and developmental stages. This observation raises the questions of how TFs choose their specific binding sites in distinct cell types and what genomic features can discriminate between cell-type-specific and shared binding sites (CSSBS). The process of discriminating these binding sites is difficult for most TFs, since they have very similar cognate motifs and sequence specificities.

Over the past several decades, the identification of transcription factor binding sites (TFBS) has made great progress, with both benefitting from the development of high-throughput sequencing technologies and the advancement of computational approaches. Specifically, ChIP-seq (Furey, 2012) provides an opportunity for viewing genome-wide interactions between DNA and specific TFs; Protein Binding Microarrays (Berger *et al.*, 2006) have enabled large-scale characterization of TF–DNA binding in a high-throughput manner without considering the influence of co-factors on predicting TFBS. As a result, massive binding data provide an unprecedented opportunity to develop computational approaches to predict TFBS. MEME (Bailey *et al.*, 2006) discovered TF–DNA binding motifs by searching for repeated, ungapped sequence patterns that occur in the biological sequences. gkm-support vector machine (SVM) (Ghandi *et al.*, 2014) detected functional regulatory elements in DNA sequences by using gapped *k*-mer features and SVM. Recently, Deep Learning (DL) has achieved amazing performance in many fields, inspiring researchers to develop DL-based methods for predicting TFBS (Alipanahi *et al.*, 2015; Quang and Xie, 2016; Zhang *et al.*, 2021, 2022; Zhou and Troyanskaya, 2015). Typically, DeepBind (Alipanahi *et al.*, 2015) and DeepSea (Zhou and Troyanskaya, 2015) applied convolutional neural network (CNN) to accurately predict diverse molecular phenotypes, including TF binding from DNA sequences. For better predicting cell-type-specific binding sites, several computational algorithms have been developed by combining DNA sequences with chromatin accessibility and histone modifications (Keilwagen *et al.*, 2019; Li *et al.*, 2019; Quang and Xie, 2019). However, either traditional methods or DL-based methods focus on distinguishing binding sites from non-binding sites in distinct cell types. This type of task (TFBS prediction) is relatively simple as the sequence specificities of binding and non-binding sites are often significantly different, so the methods that even use DNA sequences alone can achieve impressive performance. On the contrary, the task of predicting CSSBS is more difficult and remains a lot of challenges due to similar sequence specificities between CSSBS.

A review article (Srivastava and Mahony, 2020) discussed the various sequence and chromatin determinants of cell-type-specific TF-binding specificity and identified the current challenges and opportunities associated with computational approaches to characterize, impute and predict cell-type-specific TF-binding patterns. As far as we know, computational approaches are rarely proposed to systematically study cell-type-specific and shared binding mechanisms. The most relevant research (Arvey *et al.*, 2012) analyzed hundreds of ChIP-seq datasets to explore the contributions of DNA sequence signal, histone modifications and DNase accessibility to cell-type-specific binding, and proposed a discriminative framework for learning DNA sequences and chromatin signals that predict cell-type-specific TF binding. This work trained an SVM-based model that uses flexible *k*-mer patterns to capture DNA sequence signals more accurately than traditional motif approaches, and used SVM spatial chromatin signatures to model local histone modifications and DNase accessibility, concluding that DNase accessibility can explain cell-type-specific binding for many factors. Another relevant work (Gertz *et al.*, 2013), focusing on studying cell-type-specific and shared binding specificities, constructed an integrative approach to study estrogen receptor α (ER) through the integration of experimental measurements of TF binding, chromatin accessibility, DNA methylation and messenger RNA (mRNA) levels, finding that ER exhibits two distinct modes for type-specific and shared binding sites; however, this study is limited to a few nuclear receptors and does not provide a computational approach for directly discriminating CSSBS.

In this article, we present an XGBoost-based model for predicting and characterizing CSSBS through the integration of multiple types of features separately characterizing sequence specificities, cooperative interactions with co-factors, cell-type-specific chromatin landscapes and 3D chromatin interactions. In light of the successful application of DL in many fields, we also present a CNN-based model for predicting CSSBS through the integration of DNA sequences and chromatin accessibility signals. Specifically, (i) we collected peaks and filtered alignments of 10 different binding factors from the GM12878 and K562 cell lines, and then utilized DESeq2 (Love *et al.*, 2014) to differentially select GM12878-specific, K562-specific and their shared binding sites; (ii) we applied MEME (Bailey *et al.*, 2006) to separately analyze the sequence preferences for these categorized binding sites, and ran LSGKM (Lee, 2016) to explain the difficulty of predicting CSSBS using DNA sequences alone; (iii) we downloaded chromatin-related landscapes, cofactor-related motifs and chromatin interaction-related data from public databases, which were then used to generate corresponding features for each binding site; (iv) on the basis of these generated features, we designed an XGBoost-based model to predict CSSBS, allowing us to better leverage multiple types of features to learn more discriminative patterns; and (v) we also designed a CNN-based model to predict CSSBS by encoding DNA sequences and chromatin accessibility signals together. Experimental results show that our proposed approaches can accurately predict CSSBS and significantly outperforms other competing methods on all tasks. Moreover, we performed an analysis of feature importance [Shapley additive explanations (SHAP) values] for cell-type-specific and shared sites, respectively, to identify independent feature contributions, observing that chromatin features play a main role in the cell-type-specific sites while motif features play a main role in the shared sites. Beyond these observations, we also explored the ability of the CNN-based method to predict CSSBS by excluding or including DNase signals, showing that chromatin accessibility can significantly improve prediction performance. Furthermore, we investigated the generalization ability of our proposed approaches to different binding factors in the same cellular environment, revealing that there exist some similar patterns of CSSBS between different binding factors in the same cellular environment.

## 2 Materials and methods

### 2.1 Data collection

ChIP-seq datasets of 10 binding factors (CEBPB, CTCF, FOS, JUNB, MAX, MYC, POLR2A, RAD21, SP1 and YY1) from the GM12878 and K562 cell lines were collected from the ENCODE Project (The ENCODE Project Consortium, 2012), following the two criteria: (i) each dataset exists both in the GM12878 and K562 cell lines; (ii) each dataset has at least two replicates. Briefly, for each dataset in each cell line, the raw reads (fastq files) of all replicates were obtained from ENCODE, and aligned to the hg19 reference genome to keep uniquely aligned reads; then, these filtered alignments (bam files) were used to generate binding peaks by using the peak calling software SPP (Kharchenko *et al.*, 2008) with default parameters. The standard data processing pipeline is available from the ENCODE DCC Github (https://github.com/ENCODE-DCC/chip-seq-pipeline2). In addition, we collected a cohort of publicly available chromatin landscapes including DNase-seq, 11 ChIP-seq histone modifications (H2A.Z, H3K4me1, H3K4me2, H3K4me3, H3K9ac, H3K9me3, H3K27ac, H3K27me3, H3K36me3, H3K79me2 and H4K20me1), MNase-seq, total RNA-seq and DNA methylation from the Roadmap Epigenomics Project (Bernstein *et al.*, 2010), jointly forming the chromatin environments of the GM12878 and K562 cell lines. We collected 5 kb-resolution Hi-C contact maps of GM12878 and K562 from another work (Salameh *et al.*, 2020) to obtain the contact information of all potential regulatory sites. All datasets used in this work are summarized in Supplementary Tables S1–S3.

### 2.2 Differential binding sites preparation

We defined differential binding sites as peaks that are significant differences between GM12878 and K562. Briefly, for each binding factor, (i) we merged all peaks from the two cell lines, and used corresponding filtered alignments (bam files) to count the number of reads from each cell line falling into each merged peak by utilizing bedtools (https://bedtools.readthedocs.io/en/latest/); (ii) optionally, we normalized the read counts by using the Equation (1) to reduce the influence of sequencing depth and peak length; (iii) we ran DESeq2 (Love *et al.*, 2014) on the normalized read counts to compute the difference significance between GM12878 and K562; and (iv) peaks with *q*-value <0.05 and log_2_ fold-change less than −2 were chosen as GM12878-specific binding sites, peaks with *q*-value <0.05 and log_2_ fold-change >2 were chosen as K562-specific binding sites, and peaks with *q*-value >0.1 and the absolute value of log_2_ fold-change <1 were chosen as shared binding sites, while the remaining peaks were abandoned. Note that, we can control the number and quality of selected peaks by modifying the *q*-value or log_2_ fold-change.

(1) $${\text{peak}}^{i}=\frac{M^{i}}{N\times L^{i}}\times 10^{9},$$

where *M*, *N* and *L* represent the mapped reads of the *i*th peak, the total mapped reads of all peaks and the length of the *i*th peak, respectively.

In this study, we applied a split-by-chromosome strategy to divide all selected peaks into training, test and validation data, where Chromosomes 8 and 16 were used as the test set, Chromosome 18 was used as the validation set while the remaining chromosomes except Y were used as the training set. This strategy is often used to avoid the possible intersections between the training and test sets. Moreover, each peak was trimmed to 600 bp length.

### 2.3 Multiple tasks

Since all peaks were differentially divided into GM12878-specific binding sites, K562-specific binding sites and their shared binding sites, correspondingly we designed three classification tasks in this study, separately named task-A, task-B and task-C. More precisely, task-A means discriminating between GM12878- and K562-specific binding sites; task-B means discriminating between CSSBS; task-C means discriminating among GM12878-specific, K562-specific and shared binding sites. Therefore, task-A and task-B are two-class classification tasks whose labels are represented by 0 and 1 while task-C is a three-class classification task whose labels are represented by 0, 1 and 2. It is worth noting that task-B is the primary task discussed in this article, corresponding to the task of predicting CSSBS.

For evaluating the performance of our proposed methods on task-A and task-B, the area under the receiver operating characteristic curve (AUC) and area under the precision-recall curve (PRAUC) were adopted; whereas for evaluating the performance of our proposed methods on task-C, the balanced accuracy allowing for imbalanced data and macro *F*1-score suitable for multi-class problems were adopted.

### 2.4 Feature generation

Previous works (Arvey *et al.*, 2012; Srivastava and Mahony, 2020) have shown that cell-type-specific binding is in large part determined by TF’s intrinsic sequence preferences, cooperative interactions with co-factors, cell-type-specific chromatin landscapes and 3D chromatin interactions. Therefore, we considered four types of feature sets in this article, including sequence features, motif features, chromatin features and chromatin interaction features.

#### 2.4.1 Sequence features

Considering that sequence-based features play an important role in identifying TFBS, we applied two different ways to generate sequence features. More precisely, (i) a more concise version of gapped *k*-mer frequency vectors (gkm-fvs), which significantly reduces the dimension of the original gkm-fvs were directly yielded by using the method (Cao and Zhang, 2020) with default parameters, resulting in a total of 7680 features; or (ii) CNN-based sequence features were yielded from the outputs of the last convolutional layer in a CNN model pre-trained on the training set, resulting in a total of 500 features.

#### 2.4.2 Motif features

Firstly, we ran MEME on each binding factor’s dataset to identify motifs that are directly relevant to their binding sites, and then collected physically- or functionally interacting co-factors with high confidence scores (0.8 in this article) through the protein–protein-interaction network (PPI) (Szklarczyk *et al.*, 2021). Secondly, we merged all relevant factors, filtered those whose motifs do not exist in the JASPAR database (Castro-Mondragon *et al.*, 2022), and acquired the corresponding positional weight matrices (PWMs) of the remaining factors. Finally, we scored DNA forward and reverse-complement sequences by the summation of the product of PWMs and sequence matrices encoded by one-hot, and then selected the top three scores as the motif features. Since each binding factor has a different number of relevant factors, its motif features have a total of *n*×3, where *n* denotes the number of relevant factors.

#### 2.4.3 Chromatin features

Fifteen types of chromatin landscapes across the GM12878 and K562 cell lines were used in this article. For each chromatin landscape, we calculated the minimum, mean and maximum signal values of each peak (600 bp length) representing the overall distribution of signals around the peak. Furthermore, we computed the difference of each chromatin landscape between GM12878 and K562 in terms of the minimum, mean and maximum signal values. Therefore, a total of 135 (2×15×3 + 15×3) chromatin features were generated.

#### 2.4.4 Chromatin interaction features

Hi-C sequencing technology quantifies contacts between all possible pairs of genomic loci making it possible to detect loops at kilobase resolution, thereby Hi-C contact maps of GM12878 and K562 at 5 kb resolution were downloaded from public databases. Briefly, we first selected the top 20 regions interacting with each peak by the normalized contact counts, and then recorded the counts and distances between the peak and its top 20 interacting regions; we calculated the minimum, mean and maximum values of the counts and distances, and computed the difference of these values between GM12878 and K562, resulting in a total of 18 (2×6 + 6) chromatin interaction features.

At last, the four types of feature sets were concatenated into vectors as the inputs to the XGBoost-based model described in the next section.

### 2.5 Models construction

In this work, we constructed two models for computational prediction and characterization of CSSBS. One is based on Ensemble Learning and another is based on DL.

#### 2.5.1 XGBoost classifier

XGBoost (Chen and Guestrin, 2016) is an optimized distributed gradient boosting library designed to be highly efficient, flexible and portable, which implements machine-learning algorithms under the Gradient Boosting framework for classification and regression tasks. In an XGBoost model, boosted trees are added into the model by optimizing the loss function from previous trees. For each binding dataset, we trained an XGBoost model using the four types of feature sets described above, and employed a grid-search strategy to select the best parameters from a fixed set of parameters (e.g. ‘max depth’: {3, 5, 6, 7, 8}; ‘learning rate’: {0.001, 0.1, 0.3, 0.5, 1}); furthermore, we set the maximum number of iterations to 1000 and applied a validation-based early stopping strategy; in the end, the final model for this dataset was re-trained using the best parameters.

#### 2.5.2 CNN classifier

Since CNN is suitable for directly dealing with DNA sequences, each peak is transformed into an *L*×4 matrix, where *L* denotes the length of this peak with one-hot format (A=[1, 0, 0, 0], C=[0, 1, 0, 0], G=[0, 0, 1, 0] and T=[0, 0, 0, 1]). For accurately predicting CSSBS, we integrated DNase signals of GM12878 and K562, the difference of DNase signals between GM12878 and K562, and the sequence matrix, jointly forming an *L*×7 matrix. The CNN-based model is composed of three convolutional blocks in which each block is made up of a convolutional layer, a ReLU layer, a max-pooling layer as well as a dropout layer, and an output block which is made up of a fully connected layer followed by a ReLU layer, a dropout layer and a fully connected layer followed by a sigmoid layer (for task-A and task-B) or a softmax layer (for task-C).

Allowing for the issues that the initialization of weights and the selection of hyperparameters may influence the overall performance of the CNN-based model, we repeatedly warmed up the CNN classifier by randomly initializing weights and searching for the best hyperparameters from a fixed set (e.g. ‘learning rate’: {0.01, 0.001, 0.0001}). Then, we re-trained the best-performing CNN model, which stores the best initialization values and hyperparameters using the training set. The cross-entropy loss was used to train the CNN model, and optimized by the Adam optimization algorithm with a batch size of 300. All details about setting parameters can refer to our released code.

### 2.6 Competing methods

As far as we know, computational approaches for predicting cell-type-specific and shared sites are rarely proposed. The most relevant work (Arvey *et al.*, 2012) proposed an SVM-based discriminative framework for learning DNA sequence and chromatin signals that predict cell-type-specific TF binding, but it still focused on classifying binding and non-binding sites. In order to evaluate the performance of our proposed methods, we chose several kinds of representative methods as the baselines, which are widely used in the task of cell-type-specific TFBS prediction.

**LSGKM**: is an improved version of gkm-SVM for large-scale datasets, which provides further advanced gapped *k*-mer-based kernel functions, resulting in considerably higher accuracy than gkm-SVM. Moreover, since gapped *k*-mer features have been proved to be better than *k*-mer features (Ghandi *et al.*, 2014), we did use gapped *k*-mer features instead of *k*-mer features in this study.

**CNN**: is the designed CNN-based model excluding chromatin accessibility (DNase signals) in this study (see Section 2.5).

**Logistic regression (LR):** is a linear model for binary classification by default, but the binary case can be extended to classes leading to the multinomial LR.

**SVC:** is an SVM-based method capable of performing binary and multi-class classification, which chooses ‘RBF’ as the kernel function for learning non-linearity and uses the ‘one-vs-one’ decision function for multi-class classification.

**Random forest (RF)**: is a typical method of Ensemble Learning, which is composed of a diverse set of randomized decision trees, where each tree is built from a sample drawn with replacement from the training set with a random subset of features.

**Gradient boosted decision trees (GBDT)**: is a generalization of boosting to arbitrary differentiable loss functions, supporting both binary and multi-class classification.

**LightGBM**: is a highly efficient Gradient Boosting Decision Tree, supporting both binary and multi-class classification. This method is suitable for large-size and high-dimension data.

LSGKM and CNN used DNA sequences alone while the remaining competing methods took all types of features described above as input. LR, SVC, RF, GBDT and LightGBM were implemented using scikit-learn (Pedregosa *et al.*, 2011), where RF, GBDT and LightGBM adopted the same grid-search strategy as XGBoost to select the best parameters for training.

## 3 Results

### 3.1 Overview of our proposed approaches for predicting CSSBS

**Fig. 1.**
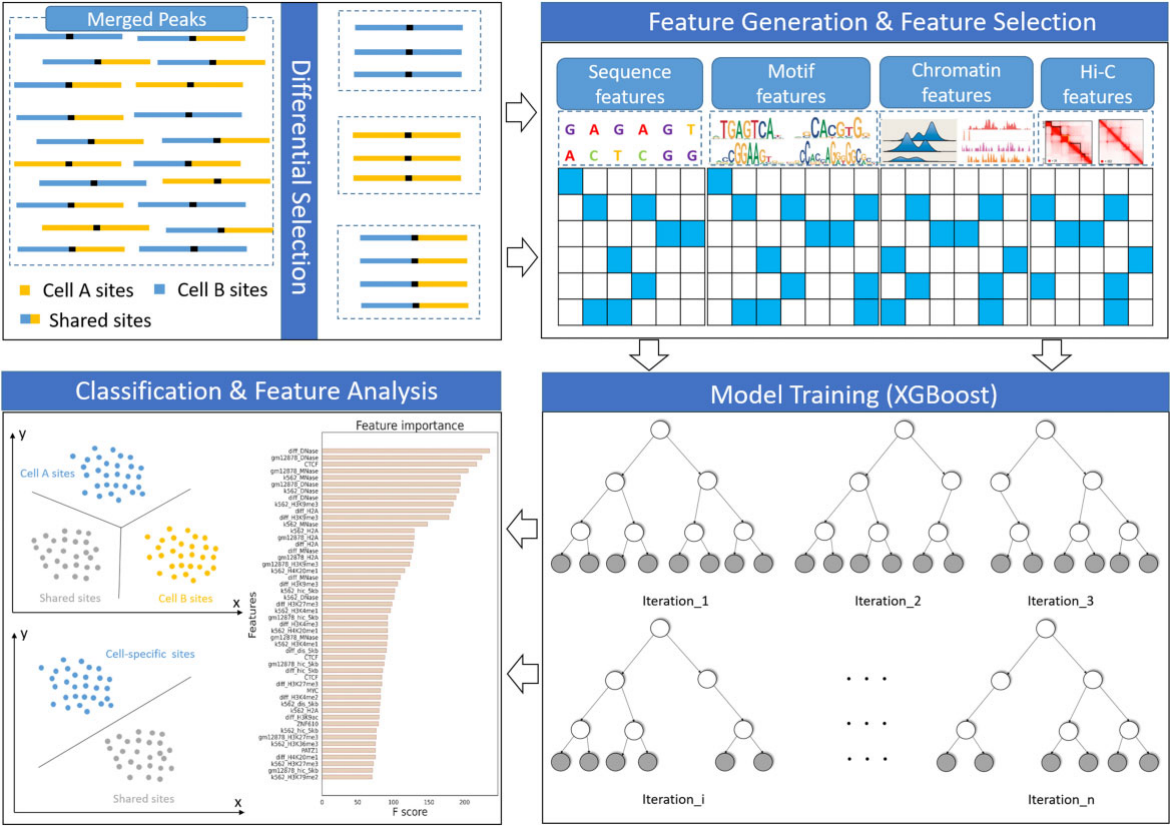
Schematic overview of the XGBoost-based model. After selecting differential binding sites, the XGBoost-based model takes sequence features, motif features, chromatin features and chromatin interaction features as inputs and outputs the probabilities of labels. The model was used to discriminate between GM12878-specific and K562-specific binding sites (task-A), discriminate between CSSBS (task-B) and discriminate among GM12878-specific, K562-specific,and shared binding sites (task-C)

As we know, a great diversity of algorithms for TFBS prediction have achieved impressive performance even using DNA sequences alone, but they mainly focus on distinguishing binding sites from non-binding sites and rarely pay attention to predicting CSSBS. Furthermore, the task of discriminating CSSBS is thought to be more difficult than the task of TFBS prediction, as CSSBS may have similar sequence specificities while binding and non-binding sites show significantly different sequence specificities (discussed in Supplementary Text S1). To accomplish the task of discriminating CSSBS, we first collected ChIP-seq datasets of 10 binding factors, including TF, cohesion, cofactor and RNA polymerase complex, from the GM12878 and K562 cell lines, and selected cell-type-specific and shared binding peaks by utilizing DESeq2and then developed an XGBoost-based method and a CNN-based method to predict and characterize these differential binding peaks. In the XGBoost-based model (Fig. 1), four types of feature sets including sequence features, motif features, chromatin features and chromatin interaction features were first generated, and then XGBoost took these features as input and output the probabilities of classes. In the CNN-based model (Supplementary Fig. S2), one-hot vectors derived from DNA sequences and DNase accessibility signals were first encoded together, and then CNN took these features as input and output the probabilities of classes. For the sake of simplicity, our proposed methods are abbreviated as XGBoost and CNN-plus, respectively, in the subsequent sections.

**Fig. 2.**
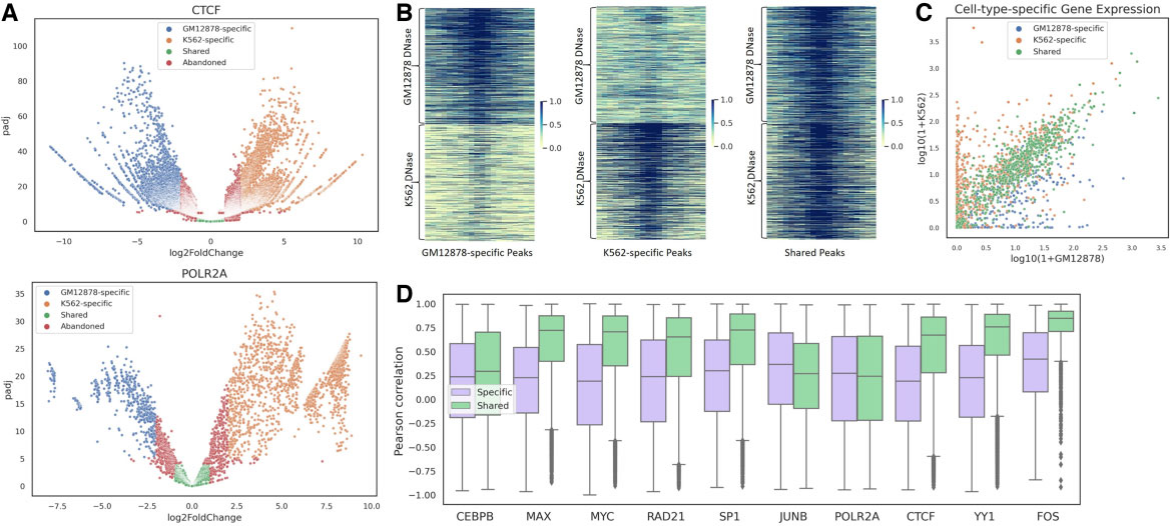
An analysis of cell-type-specific and shared binding peaks. (**A**) The distribution of binding peaks for CTCF and POLR2A where those with *q*-value <0.05 and log2 fold-change less than −2 are viewed as GM12878-specific binding sites, those with *q*-value <0.05 and log2 fold-change >2 are viewed as K562-specific binding sites and those with *q*-value >0.1 and absolute value of the log2 fold-change <1 are viewed as shared binding sites, while those labeled by red color represent abandoned peaks. Note that, we can control the number and quality of selected peaks by modifying the *q*-value and the log2 fold-change. (**B**) The visualization of CTCF DNase signals between GM12878 and K562 for GM12878-specific, K562-specific and shared binding peaks. (**C**) The cell-type-specific gene expression (RNA-seq) of genes closest to POLR2A binding peaks between GM12878 and K562. (**D**) The Pearson correlation of DNase signals between cell-type-specific and shared binding peaks across 10 binding factors (A color version of this figure appears in the online version of this article)

Figure 2A displays the distribution of binding peaks for CTCF and POLR2A (other binding factors can be found in Supplementary Fig. S3), in which GM12878-specific, K562-specific, shared and abandoned peaks are labeled by different colors, implying that we can control the quality and number of selected peaks by adjusting the *q*-value and log2 fold-change. To validate the reasonability of these peaks, for CTCF, we extracted the DNase signals of GM12878 and K562 around each peak and plotted the heat maps of them. As shown in Figure 2B, the DNase signals of GM12878 are enriched in the GM12878-specific peaks and the DNase signals of K562 are enriched in the K562-specific peaks, while the DNase signals of GM12878 and K562 are both enriched in the shared peaks. For POLR2A, since it is the largest subunit of RNA polymerase II responsible for transcriptional activities, we searched for the nearest gene to each peak and extracted its corresponding gene expression level from RNA-seq. As shown in Figure 2C, in general, the genes for cell-type-specific peaks are differentially expressed (distributed on both sides) while the genes for shared peaks are similarly expressed (clustered on the diagonal line). Note that not all peaks of POLR2A are involved in synthesizing mRNA in the process of gene transcription, e.g. enhancers can also be transcribed into eRNA (Andersson and Sandelin, 2020), so a few points are crossly distributed. Besides, we computed the Pearson correlation of DNase signals between GM12878 and K562 for cell-type-specific and shared binding peaks from 10 binding factors. As shown in Figure 2D, the Pearson correlation of shared binding peaks is almost all higher than that of cell-type-specific binding peaks. These observations demonstrate that the way of selecting cell-type-specific and shared binding peaks is reasonable and feasible.

### 3.2 Chromatin accessibility enhances CNN’s ability to predict CSSBS

**Fig. 3.**
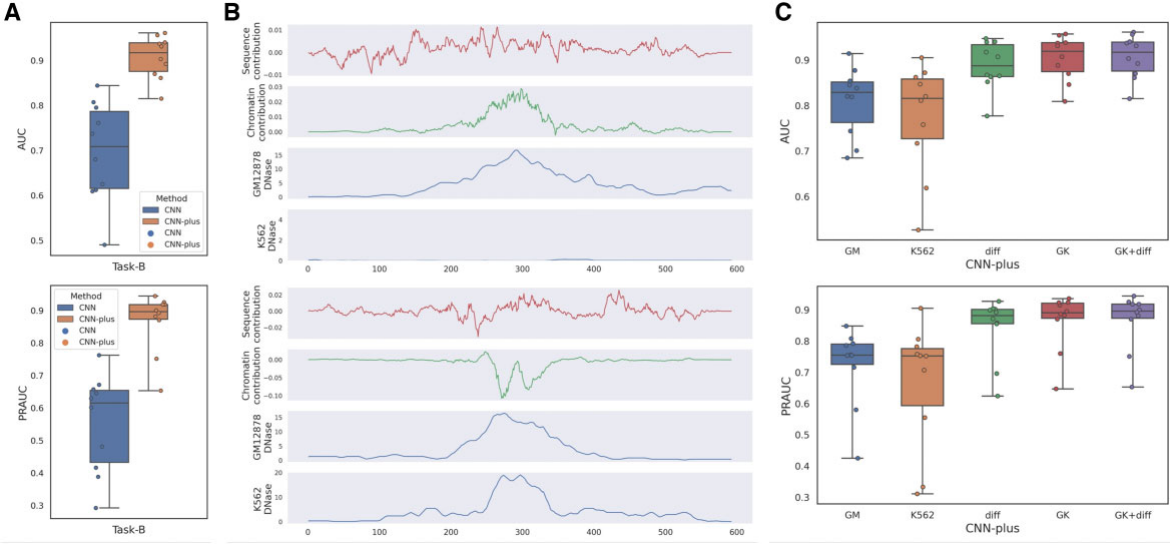
(**A**) The performance comparison of CNN and CNN-plus on predicting CSSBS (task-B). (**B**) The nucleotide-resolution contributions of DNA sequences and chromatin accessibility coupled with cell-type-specific DNase signals in which the top panel corresponds to a cell-type-specific binding site and the bottom panel corresponds to a shared binding site. (**C**) The performance comparison of CNN-plus on predicting CSSBS (task-B) under different combinations of DNase-related features, where diff. denotes the difference of DNase signals between GM12878 and K562, and GK represents a combination of GM12878 and K562 DNase signals

Without the cell-type-specific information on chromatin accessibility, those DL-based models that rely only on DNA sequences cannot distinguish the diverse TF-binding profiles across different cell types. Recent methods, such as FactorNet (Quang and Xie, 2019) and Leopard (Li and Guan, 2021), address this problem by considering both DNA sequence and chromatin accessibility, greatly improving the prediction performance. However, they still focus on the task of discriminating between binding and non-binding sites. On the contrary, we used GM12878-specific DNase signals, K562-specific DNase signals and the difference between them as the cell-type-specific information on chromatin accessibility, together with DNA sequences to predict CSSBS. To verify the impact of the cell-type-specific chromatin accessibility on predicting CSSBS, we compared CNN with CNN-plus on task-B, where CNN-plus takes DNA sequences and cell-type-specific chromatin accessibility signals as input while CNN uses DNA sequences alone. As shown in Figure 3A, we observe that CNN-plus significantly outperforms CNN on task-B, and the average AUC and PRAUC values are improved by about 21% and 31%, respectively, strongly supporting that chromatin accessibility can enhance CNN’s ability to predict CSSBS. Moreover, we computed the contribution of each base (nucleotide) to the prediction by applying DeepLIFT (Shrikumar *et al.*, 2017), which is a method for decomposing the output prediction of a neural network on a specific input by backpropagating the contributions of all neurons in the network to every feature of the input. This method assigns contribution scores according to the difference between the activation of each neuron to its ‘reference’ activation. Therefore, to investigate the relative contributions of DNA sequences and chromatin accessibility, we designed two ‘references’ for each input where one was equal to the input whose sequence-related values were set to 0 while another was equal to the input whose chromatin-related values were set to 0. For each reference, we got a contribution matrix through DeepLIFT, and the matrix was then summed along the first dimension to yield a contribution vector. In Figure 3B, the top panel shows an example of cell-type-specific binding peaks, and the bottom panel shows an example of shared binding peaks, observing that (i) the contribution of chromatin accessibility is more enriched than the one of DNA sequences and consistent with its cell-type-specific chromatin accessibility; (ii) cell-type-specific chromatin accessibility makes a positive contribution while shared chromatin accessibility makes a negative contribution, thereby providing more discriminative features for predicting CSSBS. The above observations demonstrate that the cell-type-specific information on chromatin accessibility is very important for such cell-type-specific predictive tasks.

To further explore the effect of each type of chromatin accessibility, we conducted some relevant ablation experiments by excluding one of three chromatin DNase signals. As shown in Figure 3C, we find that (i) CNN-plus using GM12878 or K562 DNase signals alone performs not very well on predicting CSSBS; (ii) CNN-plus using the difference of DNase signals between GM12878 and K562 can achieve very impressive performance, and the average performance is improved by about 8% (AUC) and 12% (PRAUC) compared with (i); and (iii) CNN-plus using all chromatin DNase signals performs best in all experiments. Overall, a combination of the three types of chromatin accessibility is essential for accurately predicting CSSBS.

### 3.3 The overall performance of our proposed methods on the three tasks

**Fig. 4.**
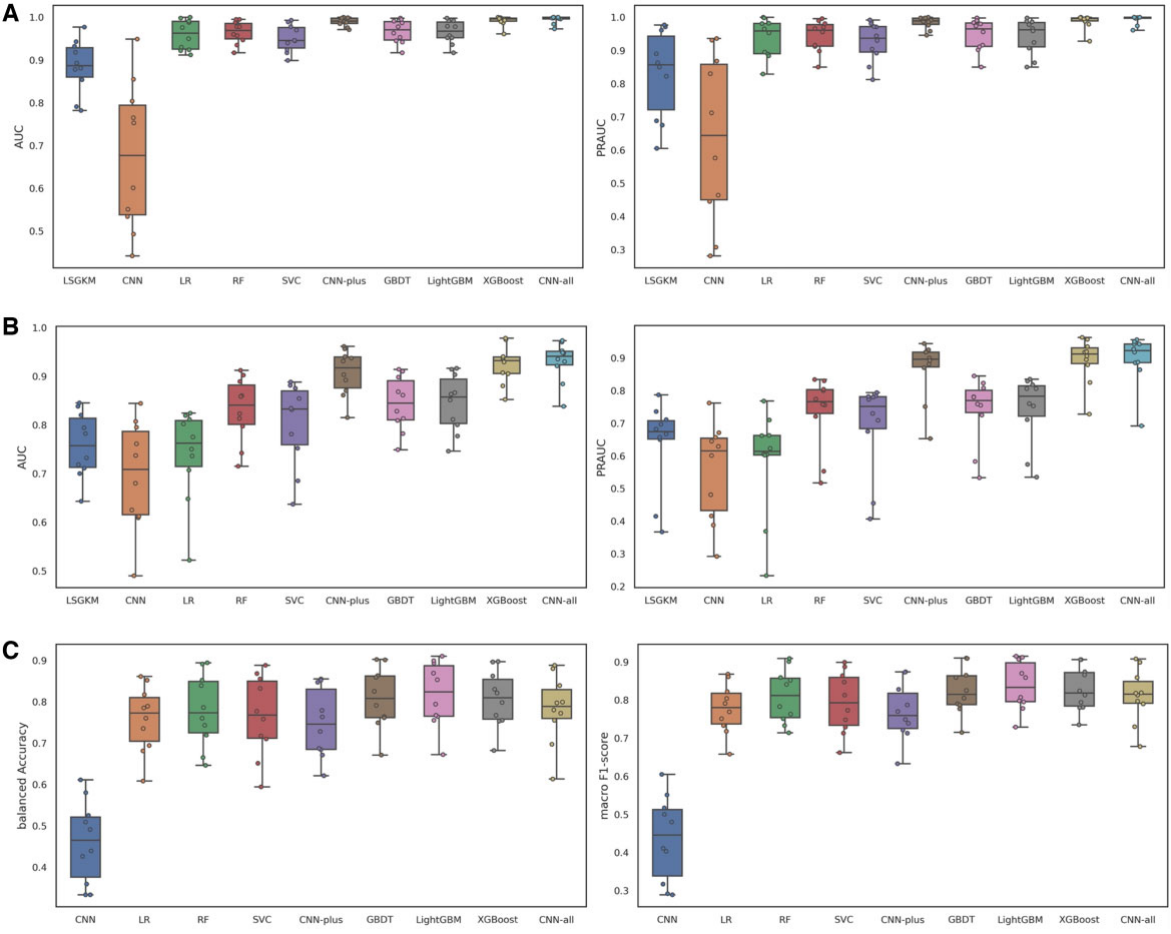
The overall performance of our proposed approaches. (**A**) The performance comparison of all methods on task-A under the AUC and PRAUC metrics. (**B**) The performance comparison of all methods on task-B under the AUC and PRAUC metrics. (**C**) The performance comparison of all methods on task-C under the balanced accuracy and macro *F*1-score metrics. The description of the three tasks please refer to Section 2.3

For XGBoost, we conducted feature ablation experiments to explore the contributions of each feature set, observing that sequence features have little effect on the performance of CSSBS prediction (as discussed in Supplementary Text S2). Hence, we retained three types of feature sets including motif features, chromatin features and chromatin interaction features derived from PPI, chromatin landscapes and Hi-C contact maps. To test the prediction performance of our proposed methods, we conducted a series of comparative experiments on the three tasks (task-A, task-B and task-C). As shown in Figure 4, LSGKM achieves high predictive accuracy and performs much better than CNN even using DNA sequences alone, implying that gapped *k*-mer encoding is a better representative of sequence specificities between cell-type-specific binding sites than nucleotide-independent one-hot encoding. Moreover, all other methods perform much better than LSGKM and CNN, demonstrating that motif features, chromatin features and chromatin interaction features are better than sequence features. Compared with LR, RF, SVC, GBDT and LightGMD, our proposed methods perform much better on the two-class classification tasks (task-A and task-B), while on the three-class classification task (task-C), XGBoost outperforms LR and RF but achieves competitive performance with GBDT and LightGMD, and CNN-plus performs worse than other methods. This observation indicates that (i) XGBoost is generally better and more robust than the competing methods; and (ii) CNN-plus is more suitable for the binary classification task. The detailed performance comparisons are displayed in Supplementary Figure S5, these performance gains show us that our proposed methods can identify discriminative features from all feature sets and learn to accurately implement classification on the three tasks.

As shown in Figure 4, the prediction performance of XGBoost is better than that of CNN-plus across the three tasks, implying that XGBoost benefits from more classification features, especially the chromatin features. To further explain the reason, we re-constructed CNN-plus by encoding DNA sequences and all chromatin landscapes (15 types) together called CNN-all, and compared it with CNN-plus and XGBoost on the three tasks. As a result, CNN-all performs much better than CNN-plus on all tasks and even performs better than XGBoost on task-A and task-B but worse than XGBoost on task-C. This observation demonstrates that (i) more chromatin features are indeed beneficial to improving the prediction performance of the CNN-based method; and (ii) the features learned by the CNN-based method are more suitable for two-class classification.

In addition, we applied SHAP (Lundberg *et al.*, 2018) to interpret the outputs of the XGBoost-based model and reveal the independent feature’s contribution to predicting CSSBS (task-B), finding that chromatin features, especially DNase-related features, play a main role in the cell-type-specific binding sites while motif features or chromatin interaction features play a main role in the shared binding sites (detailed in Supplementary Text S3).

### 3.4 Cross-factor prediction of CSSBS

**Fig. 5.**
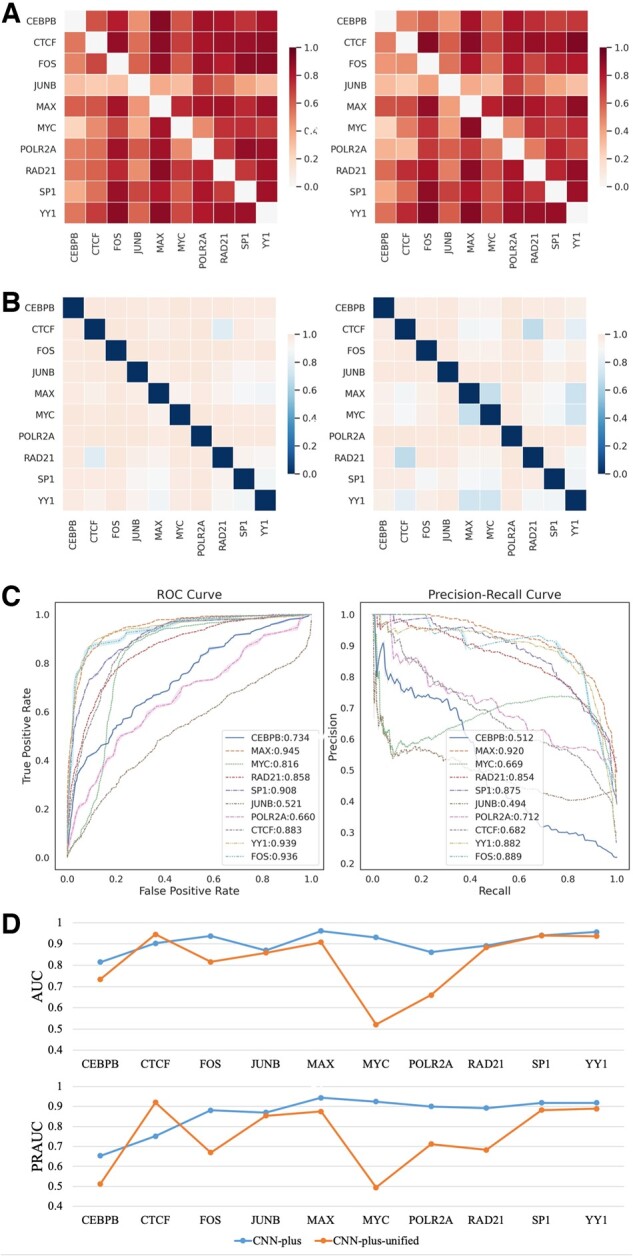
(**A**) The cross-factor performance of CNN-plus (left) and XGBoost (right) in the same cellular environment, which means that the model trained on one binding factor’s training data from GK was used to test other binding factors’ test data from GK. ‘GK’ denotes GM12878 and K562. (**B**) The Jaccard distances of cell-type-specific binding peaks between each pair-wise factor (left) and the Jaccard distances of shared binding peaks between each pair-wise factor (right). The greater the distance between binding datasets, the greater the difference between them. (**C**) The performance of the unified CNN-plus models on predicting CSSBS, where each model was trained using all training datasets except itself. (**D**) The prediction performance comparison of the unified CNN-plus and original CNN-plus models on all datasets

To evaluate the cross-factor prediction performance of our proposed approaches, we used the model trained on 1 of the 10 differential binding datasets to test the remaining 9 datasets in rotation. This allows us to study different binding factors in the same cellular environment. The overlapping peaks of different binding datasets can also influence the prediction performance since those overlapping peaks have the same chromatin environment and yield similar feature sets (specifically chromatin features). As shown in Figure 5A and B, we not only computed the cross-factor PRAUC of CNN-plus and XGBoost but also the Jaccard distances between all binding datasets. In general, we find that, in most cases, our proposed approaches perform well for cross-factor prediction of CSSBS although their Jaccard distances are much large (the average PRAUC: 0.692; the average distance: 0.94), and that CNN-plus is slightly better than XGBoost in terms of the average PRAUC (0.697 versus 0.686). For some individual cases, our approaches achieve high PRAUC (0.778 and 0.894) on the MAX and MYC binding datasets in which a large number of peaks between them are shared (small distance in Fig. 5B) since they can form a Myc-Max heterodimer that plays a very important role in cancer cells (Singh *et al.*, 2021). There is a lot of evidence that cohesin (RAD21) cooperates with CTCF to form CTCF/cohesin anchors and mediate the formation of genome-wide chromatin loops (Rudan *et al.*, 2015; Tang *et al.*, 2015), supported by their large number of shared peaks (small distance in Fig. 5B). The models trained on CTCF achieve high PRAUC values (CNN-plus: 0.858, XGBoost: 0.868) on RAD21 while the models trained on RAD21 achieve relative low PRAUC values (CNN-plus: 0.592, XGBoost: 0.741), which demonstrates that RAD21 (cohesion subunit) are not only involved in the formation of CTCF-mediated chromatin loops but also other types of chromatin loops, such as promoter-enhance interactions. The model trained on JUNB performs well on POLR2A but poorly on other datasets although the distance between them is very large (almost getting to 1), suggesting that JUNB can act as a cofactor to engage in the initialization of transcription. It is worth noting that some binding factors, such as CEBPB, FOS and POLR2A, rarely overlap with other factors but the models trained on them perform well for cross-factor prediction of CSSBS. These observations imply that there exist some similar patterns of CSSBS between different binding factors in the same cellular environment regardless of distinct sequence specificities between them, demonstrating that our proposed approaches have a good generalization ability of cross-factor CSSBS prediction. To further demonstrate it, for each binding dataset, we trained a unified CNN-plus model using the training set from all binding datasets except it and then used the trained model to measure the prediction performance on its test set. As a result, the unified CNN-plus performs well on all binding datasets (Fig. 5C) and achieves similar results to the original CNN-plus on the majority of datasets (Fig. 5D), getting an average AUC and PRAUC of 0.82 and 0.75. This observation further proves the existence of similar patterns of CSSBS between different binding factors in the same cellular environment. In the same way, we also built a unified CNN-all model for each binding dataset, observing better results and similar patterns (Supplementary Fig. S8).

## 4 Conclusions

Unlike TFBS prediction, which is aimed at distinguishing binding sites from non-binding sites, the study of computational prediction of CSSBS is less explored. Although an increasing number of computational methods have been proposed for TFBS prediction and achieved very impressive performance, these methods cannot accurately predict CSSBS when using DNA sequences alone. As our experiments show that LSGKM and CNN, two of the most popular methods, can easily realize the classification of binding and no-binding sites but fail to accurately predict CSSBS. In this article, thus we proposed two models to predict CSSBS where one is based XGBoost and another is based on CNN. Except for CSSBS prediction (task-B), we constructed other two tasks (task-A: discriminating between GM12878- and K562-specific binding sites; task-C: discriminating among GM12878-specific, K562-specific and shared binding sites) to verify the prediction performance of our proposed methods. As we expected, our proposed approaches significantly outperform other competing methods on the three tasks. Through some further experimental analysis, we observe that (i) chromatin features play a main role in the cell-type-specific binding sites while motif features play a main role in the shared binding sites, and (ii) the cross-factor prediction performance of CNN-plus is better than that of XGBoost, proving the existence of the similar patterns of CSSBS between different binding factors.

To further explore the superiority of the XGBoost-based model, we found that XGboost is capable of reducing redundant features meanwhile improving the prediction performance (discussed in Supplementary Text S4). Moreover, we observed that the flanking bins (referring to the contextual information) can improve the prediction performance of the XGBoost-based model, and that the central bin and the flanking bins have different important features that contribute to the prediction performance (discussed in Supplementary Text S5). On the contrary, XGBoost needs much more effort to do feature engineering while CNN-plus can achieve competitive prediction performance with only DNA sequences and chromatin accessibility signals.

## Supplementary Material

btac798_Supplementary_Data
