# Supplementary material for: Computational prediction and characterization of cell-type-specific and shared binding sites
Source: Bioinformatics. 2022 Dec 9;39(1):btac798. doi: 10.1093/bioinformatics/btac798 (PMC9825777; doi:10.1093/bioinformatics/btac798)
Supplement: btac798_Supplementary_Data [file btac798_supplementary_data.docx]

**Supplementary Materials**

**Supplementary text 1.** The difficulty of predicting cell-type-specific and shared binding sites (CSSBS) using DNA sequences alone.

**Supplementary text 2.** Feature ablation experiments for exploring the contributions of each feature set.

**Supplementary text 3.** Feature importance analysis to identify independent feature contributions.

**Supplementary text 4.** XGBoost is capable of reducing redundant features meanwhile improving the prediction performance.

**Supplementary text 5.** The contextual information of binding sites improves the prediction performance of XGBoost.

**Supplementary figure 1**. An analysis of the difficulty of predicting CSSBS using DNA sequences alone.

**Supplementary figure 2**. Schematic overview of the CNN-based model.

**Supplementary figure 3**. The distribution of binding peaks for SP1, RAD21, CEBPB, YY1, MAX, JUNB, FOS, and MYC.

**Supplementary figure 4**. The performance comparison of XGBoost on predicting CSSBS under different combinations of feature sets.

**Supplementary figure 5**. The overall performance comparison of all methods on task-A, task-B, and task-C.

**Supplementary figure 6**. Feature importance analysis for CTCF, JUNB, POLR2A, and RAD21.

**Supplementary figure 7.** Feature importance analysis for CEBPB, FOS, MAX, MYC, SP1, and YY1.

**Supplementary figure 8.** The prediction performance of the unified CNN-all models on predicting CSSBS.

**Supplementary figure 9.** The prediction performance of XGBoost on CEBPB and CTCF datasets when adopting the ratio range from 0.1 to 1 with an interval of 0.1.

**Supplementary figure 10.** The effect of context information on the prediction performance.

**Supplementary figure 11.** The important features of different bins whose importance is computed using all bins and then separately extracted by bins.

**Supplementary figure 12.** The important features of different bins whose importance is computed using the corresponding bin.

**Supplementary table 1.** The accession list of ChIP-seq datasets for 10 binding factors from the GM12878 and K562 cell lines.

**Supplementary table 2.** The accession list of chromatin landscapes from the GM12878 and K562 cell lines.

**Supplementary table 3.** The number of GM12878-specific, K562-specific, and shared binding peaks across all datasets.

**Supplementary Texts**

**Supplementary text 1. The difficulty of predicting cell-type-specific and shared binding sites (CSSBS) using DNA sequences alone**

To demonstrate the difficulty of predicting CSSBS, we conducted a preliminary analysis of motif similarity by running MEME (Bailey et al. 2006)) on GM12878-specific, K562-specific, and shared binding peaks respectively. As shown in Supplementary figure 1A (CEBPB and CTCF), the corresponding canonical motifs are all found in the GM12878-specific, K562-specific, and shared peaks respectively, and most of the identified cobinding factors are also overlapped. Furthermore, we used FIMO (Grant et al. 2011) to compare motif hits among GM12878-specific, K562-specific, shared, and non-binding peaks, finding that the ratio of motif hits to the total number of peaks and matched p-value are almost identical across the GM12878-specific, K562-specific, and shared peaks but higher than that of non-binding peaks (Supplementary figures 1B and 1C). These observations imply that the task of predicting CSSBS is more difficult than the task of predicting TFBS.

As we mentioned above, the task of TFBS prediction is to distinguish binding sites from non-binding sites, achieving significant performance even using DNA sequences alone. To quantitatively analyze the difficulty of predicting CSSBS, we compare task-A and task-B with the task of TFBS prediction (see ‘Multiple tasks’ in Methods). Briefly, for task-A, we performed LSGKM (Lee 2016) using DNA sequences derived from the GM12878-specific and K562-specific binding peaks; for task-B, we similarly performed LSGKM using DNA sequences derived from the cell-type-specific and shared binding peaks. However, the peaks for task-A or task-B were regarded as the positive set while the upstream regions of these peaks were extracted as the negative set in the task of TFBS prediction; then, we ran LSGKM on the positive and negative sets. As shown in Supplementary figures 1D and 1E, we find that (i) the overall performance of TFBS prediction is significantly better than that of task-A (the average AUC: 0.901 vs. 0.885; the average PRAUC: 0.891 vs. 0.83) and task-B (the average AUC: 0.936 vs. 0.758; the average PRAUC: 0.93 vs. 0.637), demonstrating that task-B (CSSBS prediction) is much more difficult than the task of TFBS prediction, and (ii) the performance of task-A is better than that of task-B (the average AUC: 0. 885 vs. 0.758; the average PRAUC: 0. 83 vs. 0.637), inferring that the sequence specificities between cell-type-specific binding sites are more discriminative than that between cell-type-specific and shared binding sites. Similarly, we can observe the same results when using CNN to do TFBS prediction (Supplementary figures 1F and 1G).

**Supplementary text 2. Feature ablation experiments for exploring the contributions of each feature set**

Since cell-type-specific binding is in large part determined by TF’s intrinsic sequence preferences, cooperative interactions with cofactors, cell-type-specific chromatin landscapes, and 3D chromatin interactions, four types of feature sets were generated as the inputs to XGBoost. We used the gkm-fvs or outputs of CNN to represent TF’s intrinsic sequence preferences, the motif scores from PPI to represent cooperative interactions with cofactors, 15 chromatin-related data (e.g, chromatin accessibility, histone modification, DNA methylation) to represent cell-type-specific chromatin landscapes, and the Hi-C contact maps to represent 3D chromatin interactions. To evaluate the relative importance of each feature set to the prediction performance of XGBoost, we conducted a series of ablation experiments by excluding one type of feature set from all sets. At first, we compared the effect of the gkm-fvs and CNN-based sequence features, finding that the performance of using CNN-based sequence features is better than that of using gkm-fvs (Supplementary figure 4). We think that the high dimensionality of gkm-fvs is unfriendly to XGBoost. Therefore, we used CNN-based sequence features in the subsequent ablation experiments. As shown in Supplementary figure 4, the performance of XGBoost without using sequence features is almost identical to the one using all features, meaning that sequence features have little effect on the performance of CSSBS prediction; the performance of XGBoost without using motif features or chromatin interaction features is slightly decreased while the performance of XGBoost without using chromatin features is significantly decreased, obviously indicating that chromatin features are major contributors to predicting CSSBS. These observations are also consistent with the findings in recent related works (Keilwagen et al. 2019; Li et al. 2019). Given the above observations, we retained motif features, chromatin features, and chromatin interaction features for XGBoost in the subsequent analysis or experiments.

**Supplementary text 3. Feature importance analysis to identify independent feature contributions**

The importance value of each feature was calculated by counting the number of its occurrences in all boosted trees. If a feature occurs more frequently in the nodes of multiple trees, it is more important. Through feature importance, we can further analyze the independent contribution of each feature instead of just analyzing the contribution of each feature set. Since we were mainly concerned about features of high importance, we selected the top 30 independent features to display. As shown in Supplementary figure 6A, we find that the most prominent feature is the ‘diff_DNase’ that denotes the difference of DNase signals between GM12878 and K562, demonstrating that this feature is the most important effector to predict CSSBS which is consistent with the finding in the analysis of CNN-plus, and that chromatin-related features account for the largest number of important factors among the top 30 independent features, again proving that chromatin features are major contributors to predicting CSSBS, since these features are perhaps very important for binding events (Quang et al. 2019; Keilwagen et al. 2019). In addition, for CTCF, its canonical motif feature is a very important effector ranked third since it has a long and strongly-specific motif; CTCF can act as a boundary factor often involved in forming chromatin loops (Rudan et al. 2015; Tang et al. 2015), thus chromatin interaction features (k562_hic_5kb and gm12878_hic_5kb) play an important role. For JUNB (AP-1 transcription factor subunit), some cofactors physically- or functionally- interacting with it are the main factors where FOSL1 is often combined with JUNB to form JUN-FOS heterodimers which strongly binds to the TPA-response element (Isakova et al. 2017); however, the top 30 features do not contain JUNB’s cognate motifs, which implies that JUNB’s motif features are not specific between cell-type-specific and shared binding sites. For POLR2A (RNA polymerase II subunit), except DNase features, RNA-seq features are the most important factors which represent their specific gene expression; the only motif feature among the top 30 features corresponds to TBP, which is a general transcription factor that functions at the core of the DNA-binding multiprotein factor TFIID, playing a role in the activation of eukaryotic genes transcribed by RNA polymerase II; chromatin interaction features are highly ranked since Hi-C experiments have proved that genome exists a large number of promoter-mediated chromatin loops such as promoter-promoter loops and enhancer-promoter loops. For RAD21 (cohesin complex subunit), it has been annotated as a factor without sequence specificity but can be combined with CTCF to form CTCF-mediated chromatin loops (Tang et al. 2015) or associated with MYC to control cohesin positioning and genome organization (Xiao et al. 2021), thus motif features for CTCF and MYC as well as chromatin interaction features are highly ranked. For the remaining 6 transcription factors, we can observe similar results that chromatin features are still the dominative factors, and motif features and chromatin interaction features are the important factors (Supplementary figure 7A). However, the feature importance computed by XGBoost just provides a global analysis of feature importance during the training process, and cannot give a deep insight into how each feature independently contributes to predicting CSSBS.

To alleviate the above problem, we utilized Shapley additive explanations (SHAP) (Lundberg et al. 2018) to interpret the outputs of our model and reveal the independent feature’s contribution to predicting CSSBS. Briefly, (i) we separated the test set into cell-type-specific binding sites (positive) and shared binding sites (negative) by labels; (ii) computed the SHAP values of the positive and negative sets respectively, yielding two *n*🞨*m* matrices where n and m denote the number of samples and features respectively; (iii) took the sum of each matrix along the sample direction, yielding a vector of length m; (iv) took the absolute value of the vector as the final feature importance, representing the independent contribution of each feature to predicting CSSBS; (v) selected the top 30 features with high SHAP values to display. As shown in Supplementary figure 6B, we observe that chromatin features, especially DNase-related features, play a main role in the cell-type-specific binding sites while motif features or chromatin interaction features play a main role in the shared binding sites. For CTCF, except itself, other potential cobinding TFs (e.g, ZNF610, MYC) are the main contributors to shared binding sites. For JUNB, DNase features are also the main contributors to cell-type-specific binding sites while its cobinding factor FOS is the most important contributor to shared binding sites. For RAD21, its cobinding factor CTCF plays a very important role in shared binding sites but not in cell-type-specific binding sites. Except for POLR2A, its DNase features are the main contributors to cell-type-specific binding sites while its RNA features are the main contributors to shared binding sites. Similarly, for the remaining 6 TFs, we observe the same results (Supplementary figure 7B). These observations are indeed consistent with the intuition that, cell-type-specific binding sites are characterized by significantly distinct DNase signals while shared binding sites are characterized by similar DNase signals, thus DNase signals are the main discriminator of cell-type-specific binding sites while other features such as cobinding factors or chromatin interactions are the main discriminator of shared binding sites.

**Supplementary text 4. XGBoost is capable of reducing redundant features meanwhile improving the prediction performance**

Given that some features used by the XGBoost model may be redundant, we designed two experiments to investigate the ability of XGBoost to handle the issue. Briefly, (i) XGBoost was separately trained on the training set of CEBPB and CTCT; (ii) the feature importance of all features was computed from the trained models; (iii) all features were sorted in descending order by their corresponding feature importance; (iv) the top-k features were selected as the non-redundant features, where k is determined by a ratio from {0.1, 0.2, 0.3, 0.4, 0.5, 0.6, 0.7, 0.8, 0.9, 1.0}; (v) XGBoost was re-trained on the feature-filtered training set, and then tested on the feature-filtered testing set. As shown in Supplementary figure 9, for CEBPB, when selecting the top 60% or 70% features (ratio=0.6 or 0.7), we can get a better result than that using all features (ratio=1.0); for CTCF, when selecting the top 50% features (ratio=0.5), we can also get a better result than that using all features (ratio=1.0). The above results on the two examples show that XGBoost is capable of reducing redundant features meanwhile improving the prediction performance.

**Supplementary text 5. The contextual information of binding sites improves the prediction performance of XGBoost**

To explore the effect of the contextual information of binding sites on the performance of predicting CSSBS, we applied CNN-plus and XGBoost to conduct two additional experiments. Briefly, for CNN-plus, we just expanded the original length of peaks from 600bp to 1000bp, since the convolutional layers can automatically learn the contextual information of binding sites in a sliding-window way; whereas for XGBoost, we allowed for the contextual information by expanding the original length of peaks from 600bp to 1000bp, segmenting the peaks of length 1000bp into multiple 200bp genomic bins, and then computing motif features, chromatin features, and chromatin interaction features for each bin. As shown in Supplementary figure 10A, CNN-plus with additional contextual information performs worse than CNN-plus, indicating that the contextual information of binding sites is not beneficial to CNN-plus. The result may be caused by the CNN’s intrinsic mechanism that automatically focuses on the most important features of inputs. However, we find that XGBboost with additional contextual information is better than XGBoost in terms of AUC and PRAUC, showing that the contextual information of binding sites can improve the prediction performance of XGBoost. These observations also suggest that long-range peaks are more suitable for XGBoost than CNN-plus since the inputs to XGBoost are the minimum, mean, and maximum values that characterize the overall distribution of binding peaks. To further demonstrate which ‘contextual information’ contributes to the performance improvement, we display the importance of each feature and show the top 30 features by their importance for each bin. The examples CTCF and JUNB are shown in Supplementary figure 11, for CTCF (figures A and B), we observe that the features of the central bin (bin3 containing peak summit) contribute the most to the prediction performance while the features of the flanking bins (bin1, bin2, bin3, and bin4) contribute less but are useful. Moreover, the central bin and the flanking bins show different feature importance. For example, DNase, MNase and CTCT motif are dominative in the central bin, which is consistent with the conclusion in the main text, while Hi-C, histone marks (eg., H4K20me1, H3K9me3) and co-binding motifs (e,g., POU5F1, SMAD5) are prominent in the flanking bins. For JUNB (figures C and D), we also observe similar results. Therefore, these results imply that the important features of the flanking bins are different from that of the central bin, and have a positive effect on the overall performance.

To directly observe the effect of the flanking bins (contextual information), we performed individual experiments for each bin from two examples CTCF and JUNB. Briefly, i) the peaks of length 1000bp were segmented into 5 non-overlapping 200bp genomic bins, named bin1, bin2, bin3, bin4, and bin5; ii) the same three types of feature sets, including motif features, chromatin features, and chromatin interaction features, were generated for each bin; iii) an individual experiment for each bin was performed by using XGBoost. As shown in Supplementary figure 10B, the prediction performance of the central bin (bin3) is much better than that of the flanking bins (bin1, bin2, bin3, and bin4) but worse than that of using all bins in terms of AUC and PRAUC, demonstrating that the flanking bins are beneficial to improve the prediction performance. And, Supplementary figure 12 shows the similar but more pronounced trends that are discovered in Supplementary figure 11, for CTCF (figures A and B), i) CTCF motif is more enriched in the central bin while co-binding motifs are more enriched in the flanking bins; ii) DNase is more enriched in the central bin while other chromatin features are more enriched in the flanking bins; iii) Hi-C features are more enriched in the flanking bins. For JUNB (figures C and D), we observe that i) co-binding motifs are more prominent than JUNB motif in bins 2, 3, and 4, implying its role of coactivator often cobinding with other factors; ii) the difference of DNase is prominent in the bins 2, 3, and 4, and most of histone marks are enriched in the flanking bins. In conclusion, these trends indicate that the flanking bins have different important features that contribute to the prediction performance of XGBoost. Through this analysis, we can discover important features in the central bin and the flanking bins from other binding datasets, respectively.

**References**

Bailey TL, Williams N, Misleh C, Li WW. 2006. Meme: Discovering and analyzing dna and protein sequence motifs. Nucleic acids research. 34(suppl_2):W369-W373.

Grant CE, Bailey TL, Noble WS. 2011. Fimo: Scanning for occurrences of a given motif. Bioinformatics. 27(7):1017-1018.

Lee D. 2016. Ls-gkm: A new gkm-svm for large-scale datasets. Bioinformatics. 32(14):2196-2198.

Quang, D. and Xie, X. (2019) FactorNet: a deep learning framework for predicting cell type specific transcription factor binding from nucleotide-resolution sequential data, Methods, 166, 40-47.

Keilwagen, J., Posch, S. and Grau, J. 2019. Accurate prediction of cell type-specific transcription factor binding. Genome biology, 20, 1-17.

Li, H., Quang, D. and Guan, Y. 2019. Anchor: trans-cell type prediction of transcription factor binding sites. Genome research, 29, 281-292.

Rudan MV, Barrington C, Henderson S, Ernst C, Odom DT, Tanay A, Hadjur S. 2015. Comparative hi-c reveals that ctcf underlies evolution of chromosomal domain architecture. Cell reports. 10(8):1297-1309.

Tang Z, Luo OJ, Li X, Zheng M, Zhu JJ, Szalaj P, Trzaskoma P, Magalska A, Wlodarczyk J, Ruszczycki B. 2015. Ctcf-mediated human 3d genome architecture reveals chromatin topology for transcription. Cell. 163(7):1611-1627.

Isakova A, Groux R, Imbeault M, Rainer P, Alpern D, Dainese R, Ambrosini G, Trono D, Bucher P, Deplancke B. 2017. Smile-seq identifies binding motifs of single and dimeric transcription factors. Nature methods. 14(3):316.

Xiao T, Li X, Felsenfeld G. 2021. The myc-associated zinc finger protein (maz) works together with ctcf to control cohesin positioning and genome organization. Proceedings of the National Academy of Sciences. 118(7).

Lundberg SM, Nair B, Vavilala MS, Horibe M, Eisses MJ, Adams T, Liston DE, Low DK-W, Newman S-F, Kim J. 2018. Explainable machine-learning predictions for the prevention of hypoxaemia during surgery. Nature biomedical engineering. 2(10):749-760.

**Supplementary Figures**

**
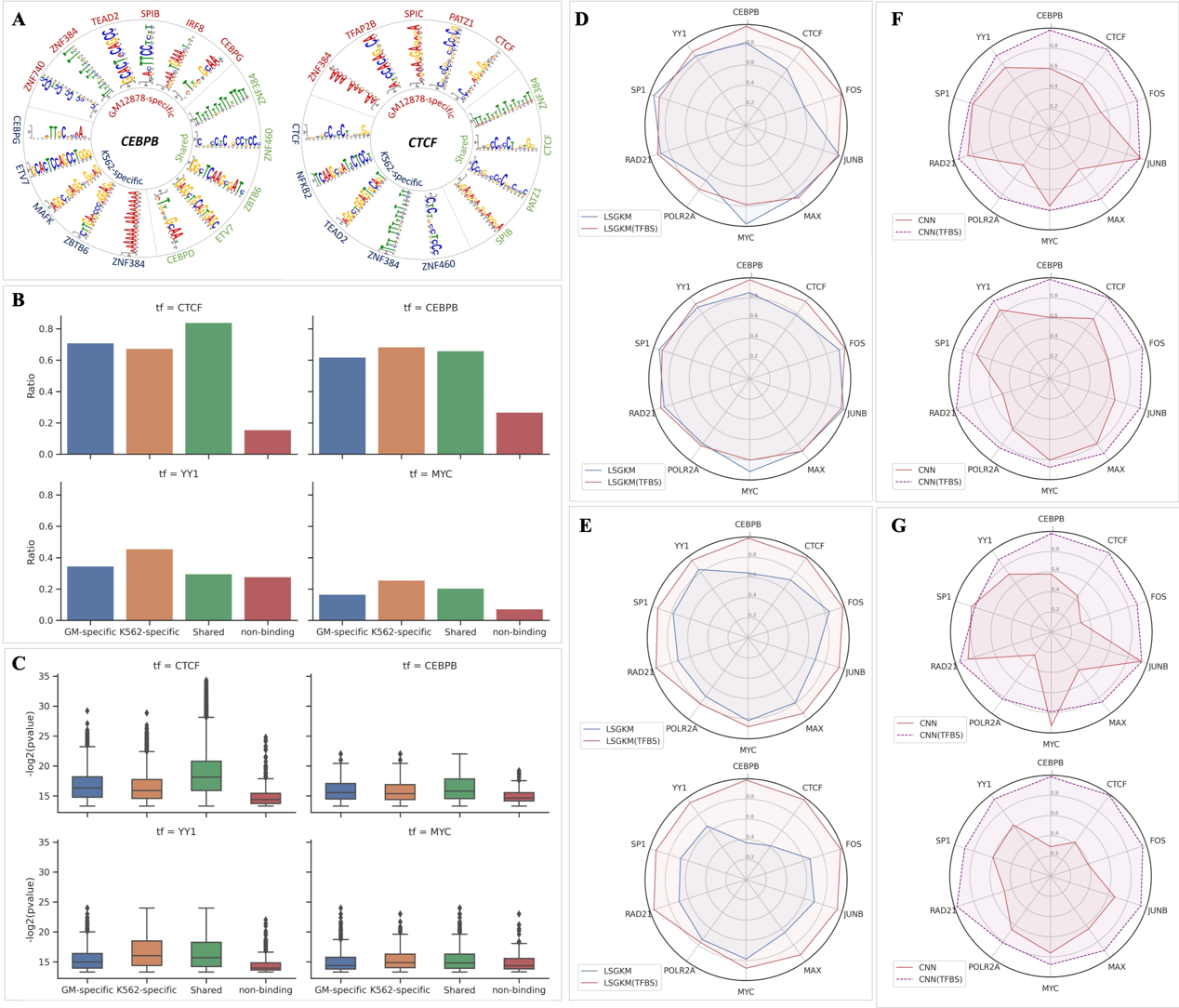
**

**Supplementary** **figure 1**. An analysis of the difficulty of predicting CSSBS using DNA sequences alone. (A) The identified motifs of GM12878-specific, K562-specific, and shared binding peaks by MEME for CEBPB and CTCF. (B) The ratio of found motif instances to all peaks across CTCF, CEBPB, YY1, and MYC. (C) The distribution of the –log2(p-value) of found motif instances across CTCF, CEBPB, YY1, and MYC. (D) The AUC and PRAUC comparison of LSGKM and LSGKM(TFBS) across all binding factors where ‘LSGKM’ means discriminating between GM12878-specific and K562-specific binding sites while ‘LSGKM(TFBS)’ means discriminating between binding sites (GM12878-specific and K562-specific binding peaks) and corresponding non-binding sites. (E) The AUC and PRAUC comparison of LSGKM and LSGKM(TFBS) across all binding factors where ‘LSGKM’ means discriminating between cell-type-specific and shared binding sites while ‘LSGKM(TFBS)’ means discriminating between binding sites (cell-type-specific and shared binding peaks) and corresponding non-binding sites. (F) The AUC and PRAUC comparison of CNN and CNN (TFBS) across all binding factors. (G) The AUC and PRAUC comparison of CNN and CNN(TFBS) across all binding factors.


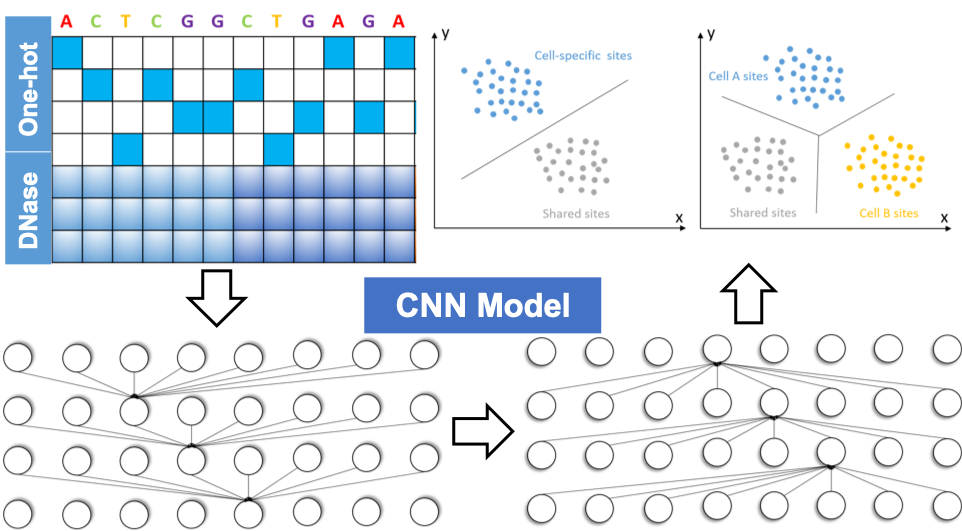


**Supplementary figure 2.** Schematic overview of the CNN-based model. After selecting differential binding sites, the CNN-based model takes DNA sequences and chromatin accessibility signals as inputs and outputs the probabilities of labels. The model was used to discriminate between GM12878-specific and K562-specific binding sites (task-A), discriminate between cell-type-specific and shared binding sites (task-B), and discriminate among GM12878-specific, K562-specific, and shared binding sites (task-C).


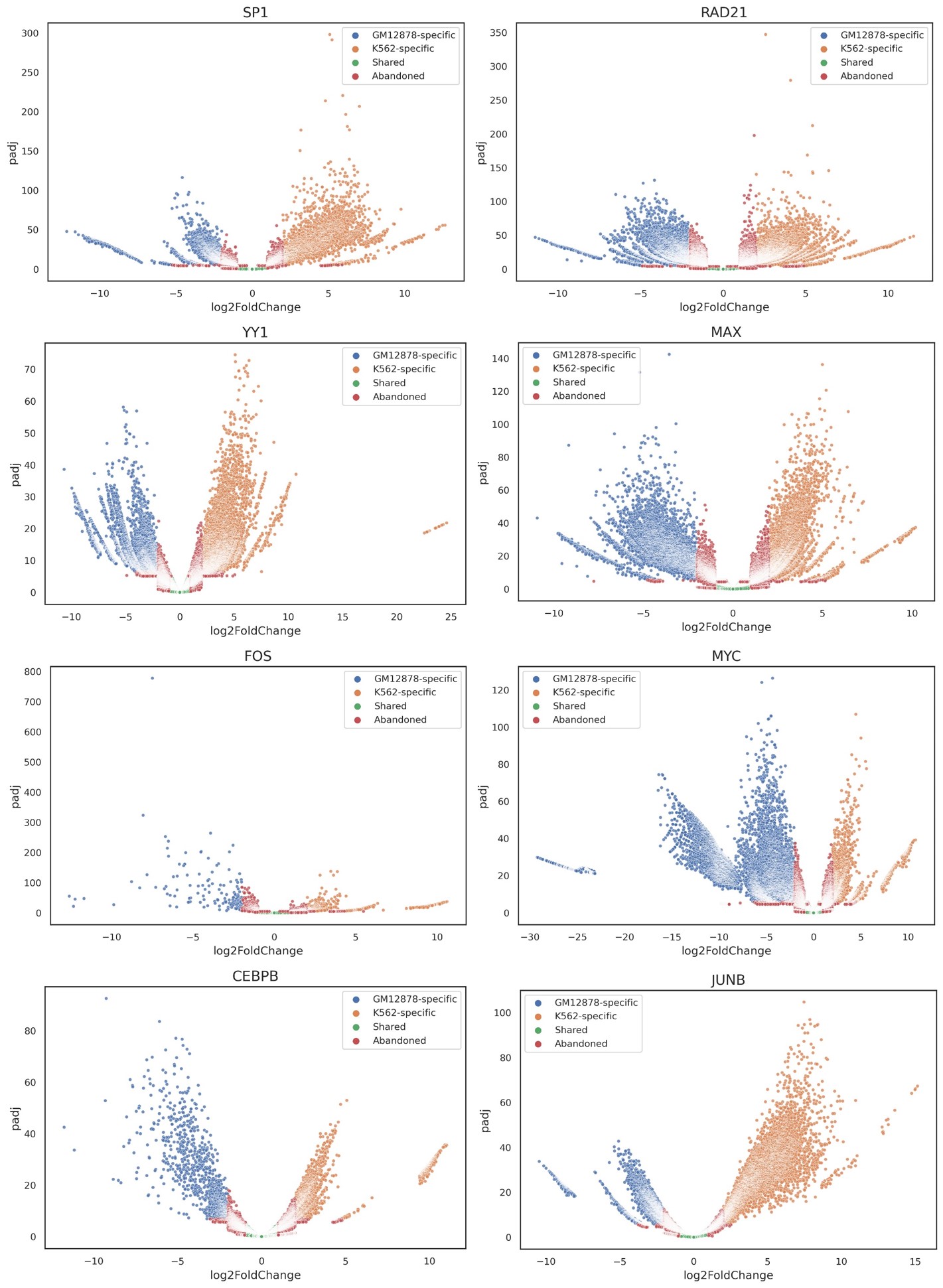


**Supplementary figure 3.** The distribution of binding peaks for SP1, RAD21, CEBPB, YY1, MAX, JUNB, FOS, and MYC, in which GM12878-specific, K562-specific, shared, and abandoned peaks are labelled by different colors.


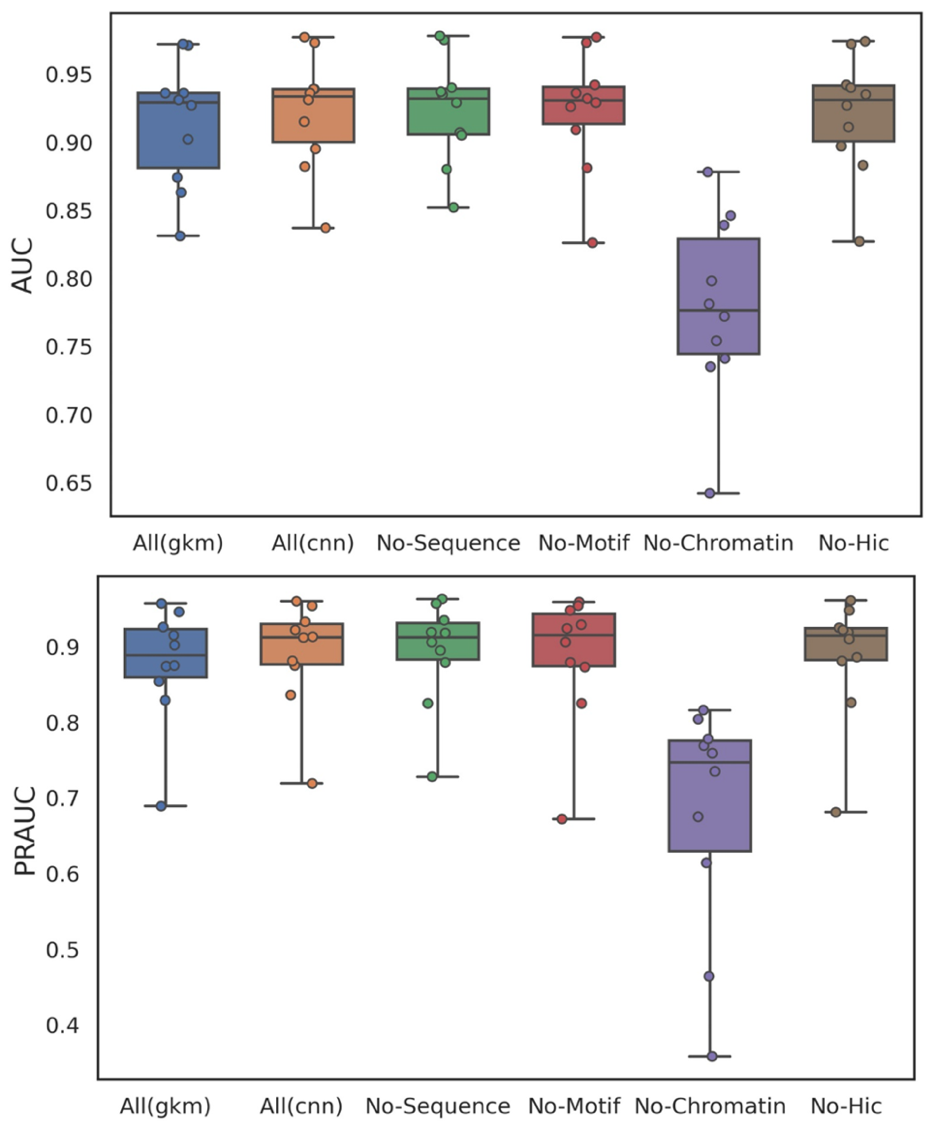


**Supplementary figure 4.** The performance comparison of XGBoost on predicting CSSBS under different combinations of feature sets.


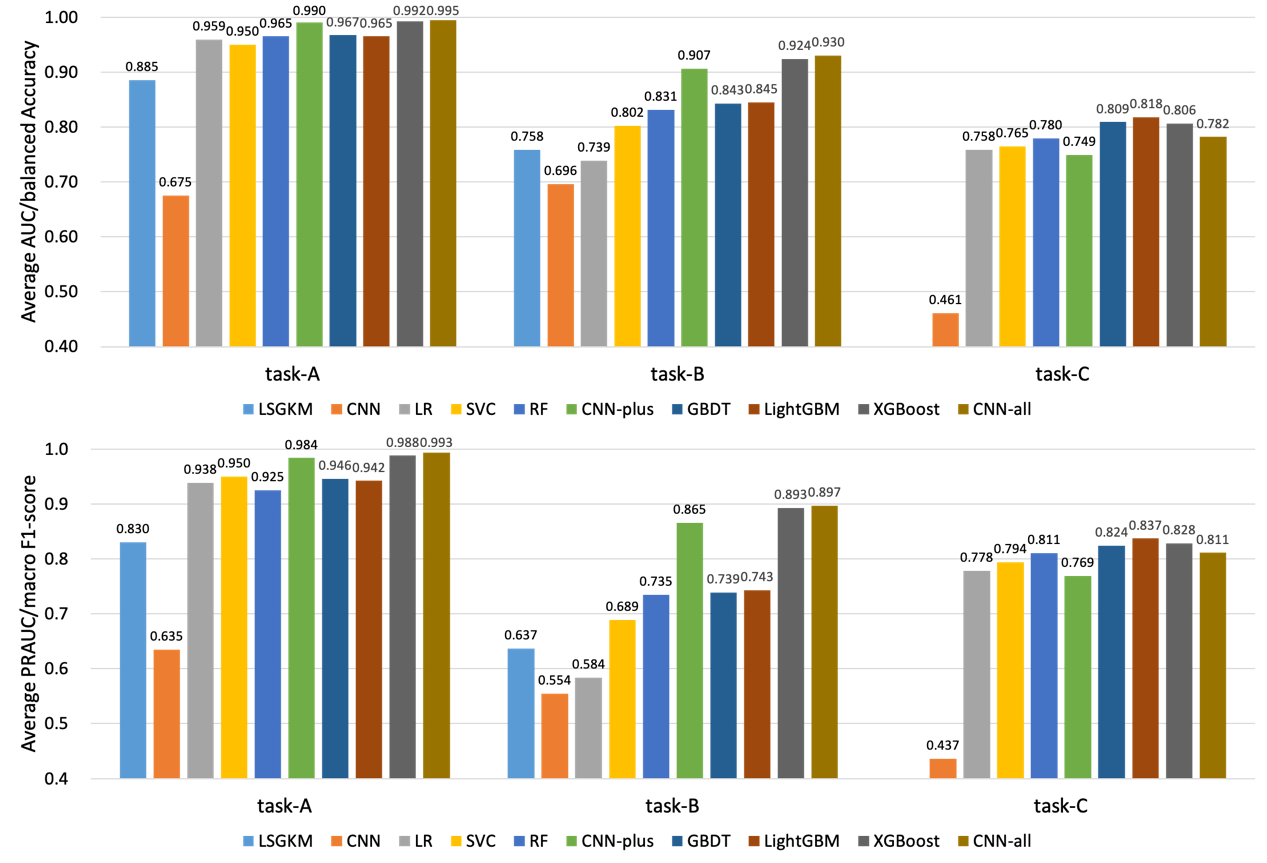


**Supplementary figure 5.** The overall performance comparison of all methods on task-A, task-B, and task-C. Compared with LSGKM, CNN, LR, RF, CNN-plus, GBDT, LightGBM, and CNN-all, for task-A, the average AUC of XGBoost is improved by about 11%, 32%, 3.3%, 4%, 3%, 0.2%, 2.5%, 2.7%, and -0.3%, respectively, and the average PRAUC of XGBoost is improved by about 16%, 35%, 5%, 6%, 4%, 0.4%, 4.2%, 4.6%, and -0.5%, respectively; for task-B, the average AUC of XGBoost is improved by about 17%, 23%, 18.5%, 12%, 9%, 2%, 8.1%, 8.4%, and -0.6%, respectively, and the average PRAUC of XGBoost is improved by about 26%, 34%, 31%, 20%, 16%, 3%, 15.4%, 15%, and -0.4%, respectively; for task-C, the average balanced accuracy of XGBoost is improved by about 33%, 4.8%, 3%, 2%, 7%, -0.3%, -1.2%, and 2.4%, respectively, and the average macro F1-score of XGBoost is improved by about 38%, 5%, 2%, 1%, 6%, 0.4%, -0.9%, and 1.7%, respectively.


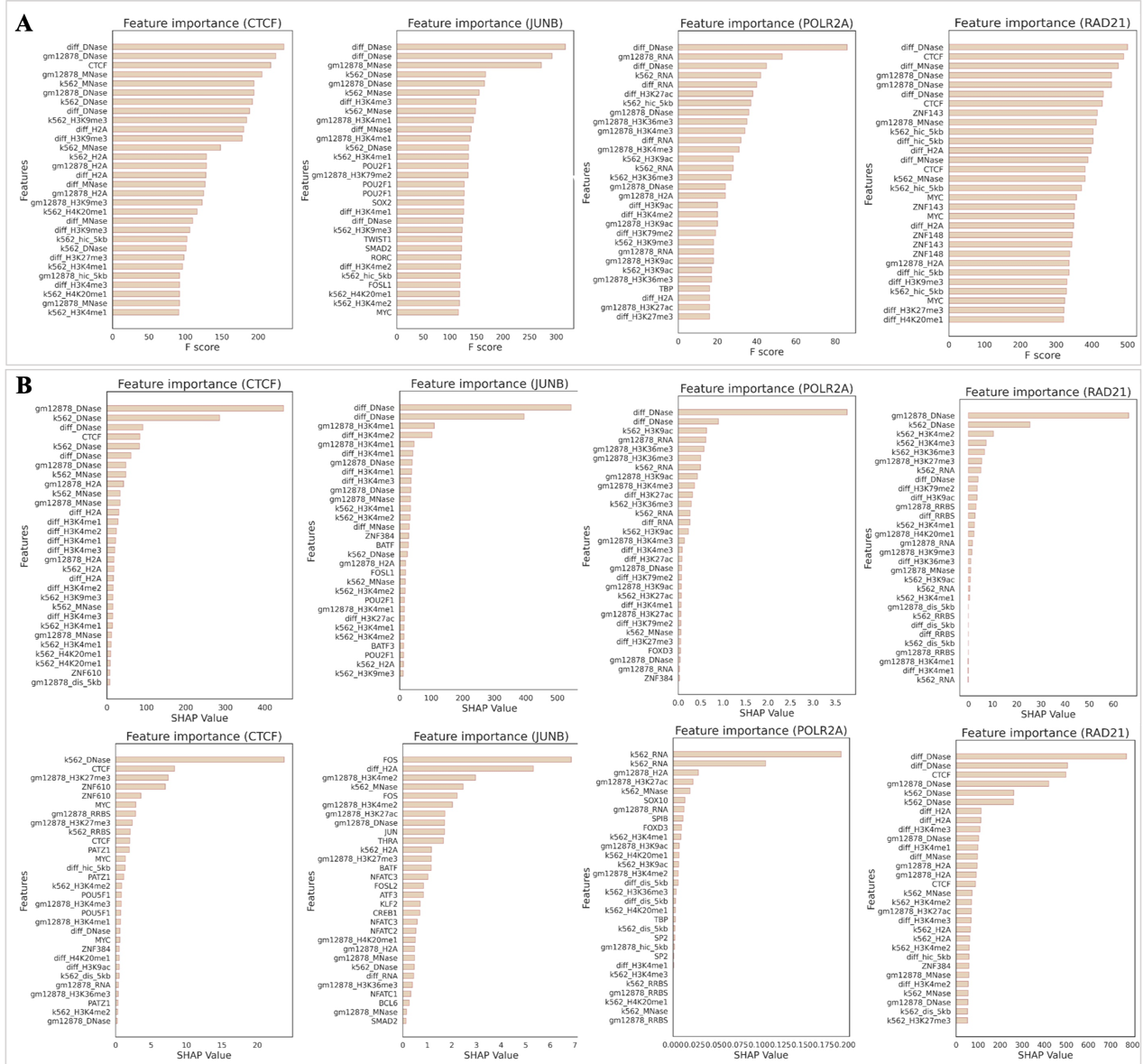


**Supplementary figure** **6**. Feature importance analysis for CTCF, JUNB, POLR2A, and RAD21. (A) The global feature importance of the top 30 features across the four factors. (B) The top 30 SHAP values of cell-type-specific (top) and shared (bottom) binding sites across the four factors. Note that the terms marked by ‘diff’ mean the difference of values between GM12878 and K562.


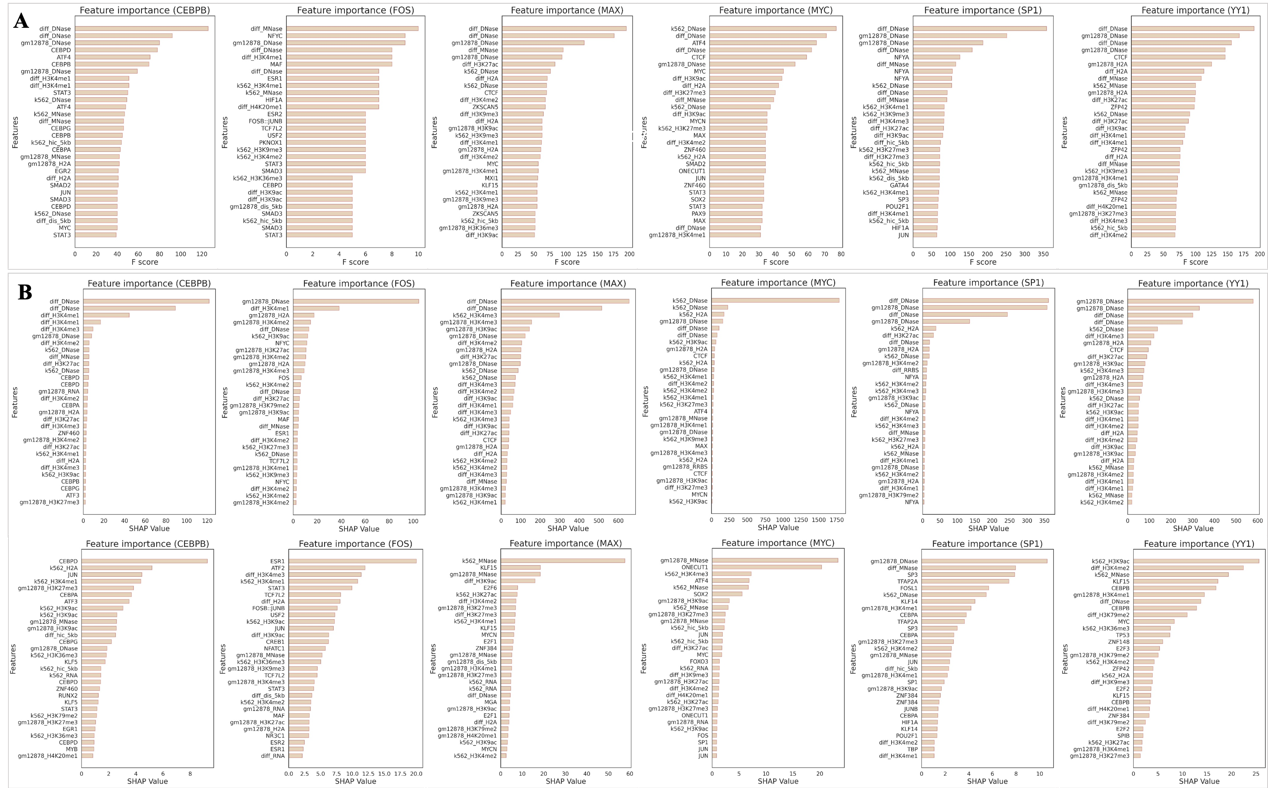


**Supplementary figure 7.** Feature importance analysis for CEBPB, FOS, MAX, MYC, SP1, and YY1. (A) The global feature importance of the top 30 features across the remaining six factors. (B) The top 30 SHAP values of cell-type-specific (top) and shared (bottom) binding sites across the remaining six factors. Note that the terms marked by ‘diff’ mean the difference of values between GM12878 and K562.


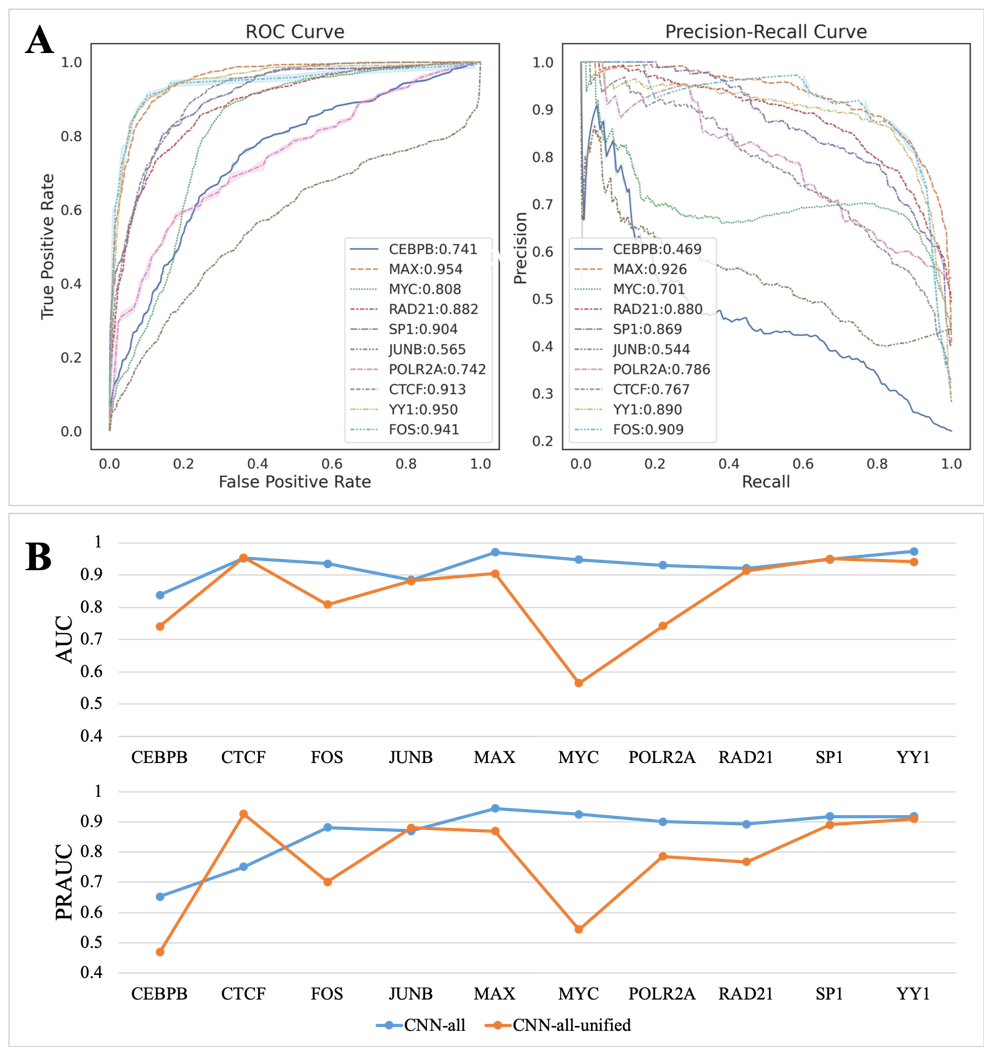


**Supplementary figure 8.** The prediction performance of the unified CNN-all models on predicting CSSBS. (A) The AUC and PRAUC values of the unified CNN-all models for all binding datasets, where each model was trained using all training datasets except itself. (B) The prediction performance comparison of the unified CNN-all and original CNN-all models on all datasets.


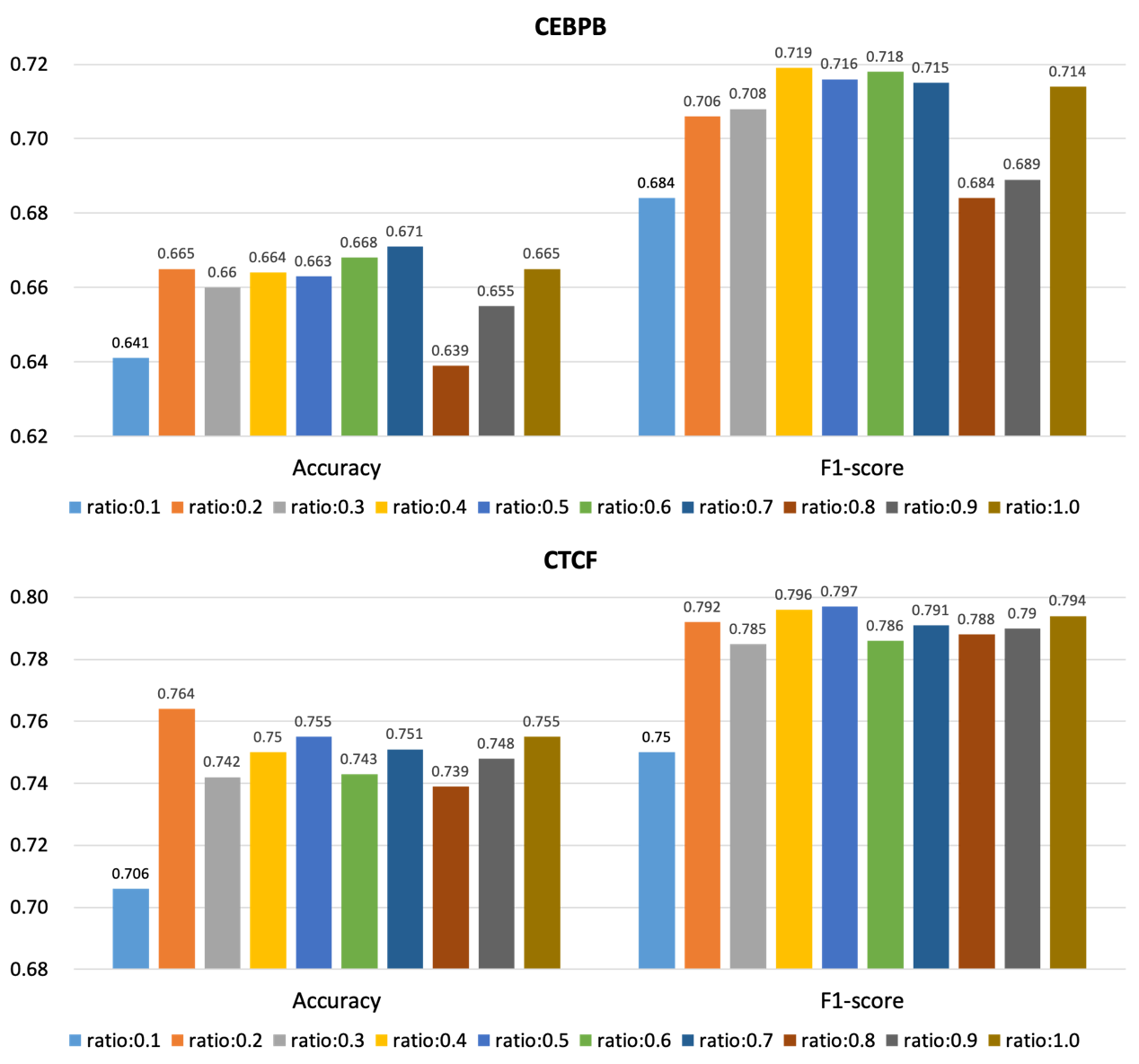


**Supplementary figure 9.** The prediction performance of XGBoost on CEBPB and CTCF datasets when adopting the ratio range from 0.1 to 1 with an interval of 0.1. The ratio 0.1 means selecting the top 10% features, and the ratio 1.0 means using all features.


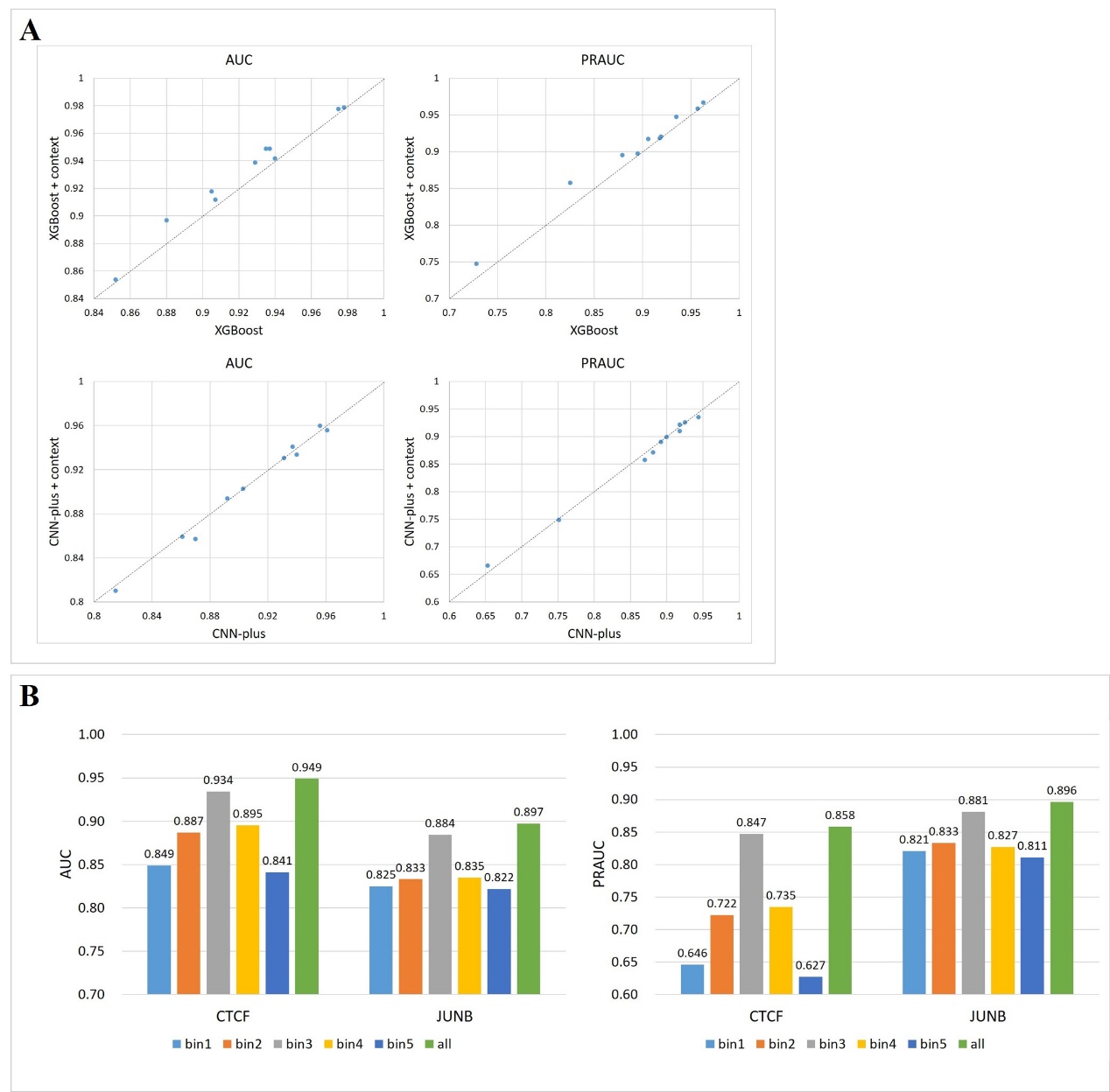


**Supplementary figure 10.** The effect of context information on the prediction performance. (A) The performance comparison of CNN-plus and CNN-plus with additional context information (top), and the performance comparison of XGBoost and XGBoost with additional context information (bottom). (B) The performance comparison of XGBoost on the central bin (bin3), the flanking bins (bin1, bin2, bin4, bin5), and all bins for CTCF and JUNB.


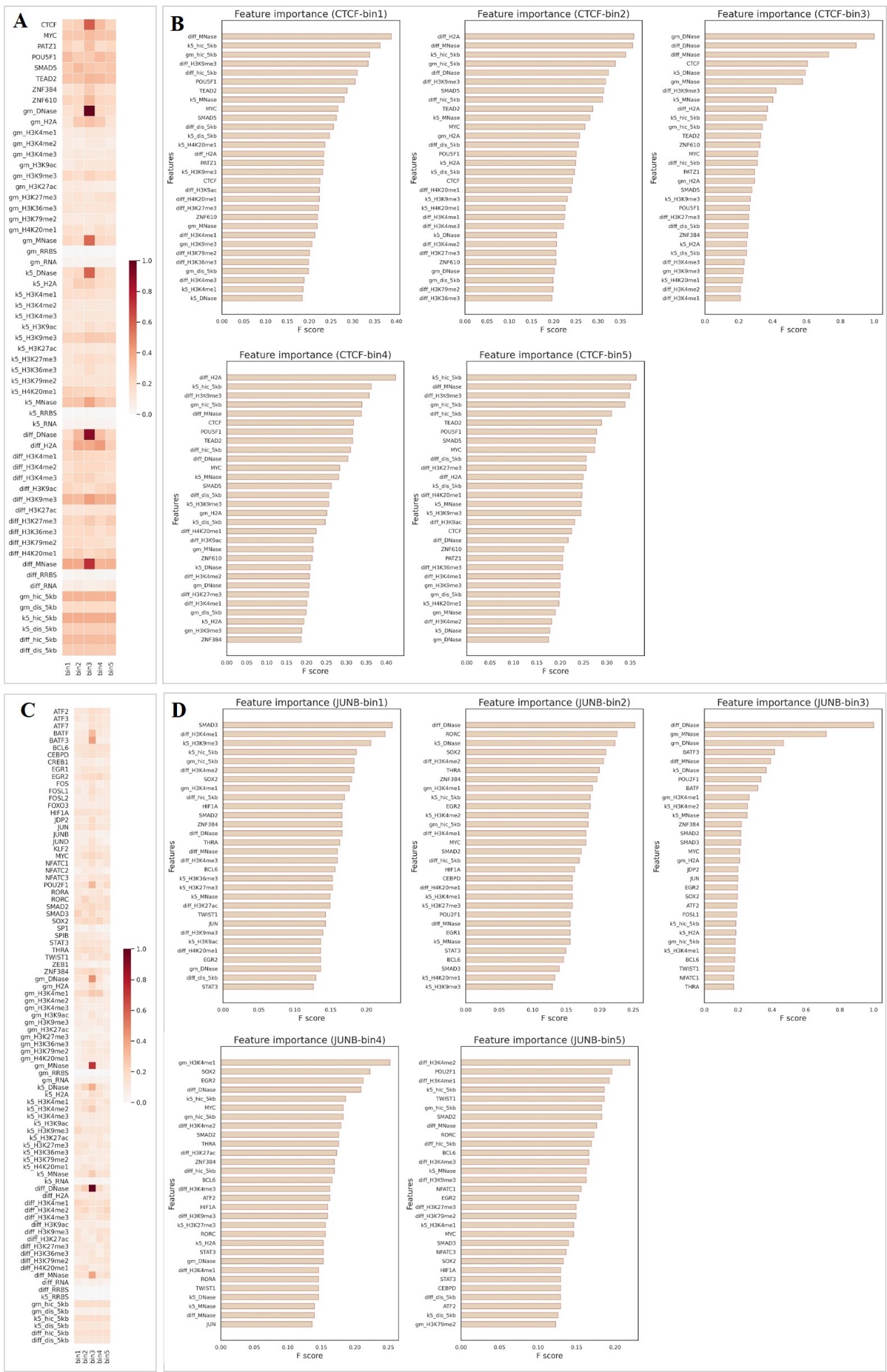


**Supplementary figure 11.** The important features of different bins whose importance is computed using all bins and then separately extracted by bins. (A) The heatmap of the feature importance of the central bin (bin3) and the flanking bins (bin1, bin2, bin4, bin5) for CTCF. (B) The top-30 important features of the central bin and the flanking bins for CTCF. (C) The heatmap of the feature importance of the central bin (bin3) and the flanking bins (bin1, bin2, bin4, bin5) for JUNB. (D) The top-30 important features of the central bin and the flanking bins for JUNB.


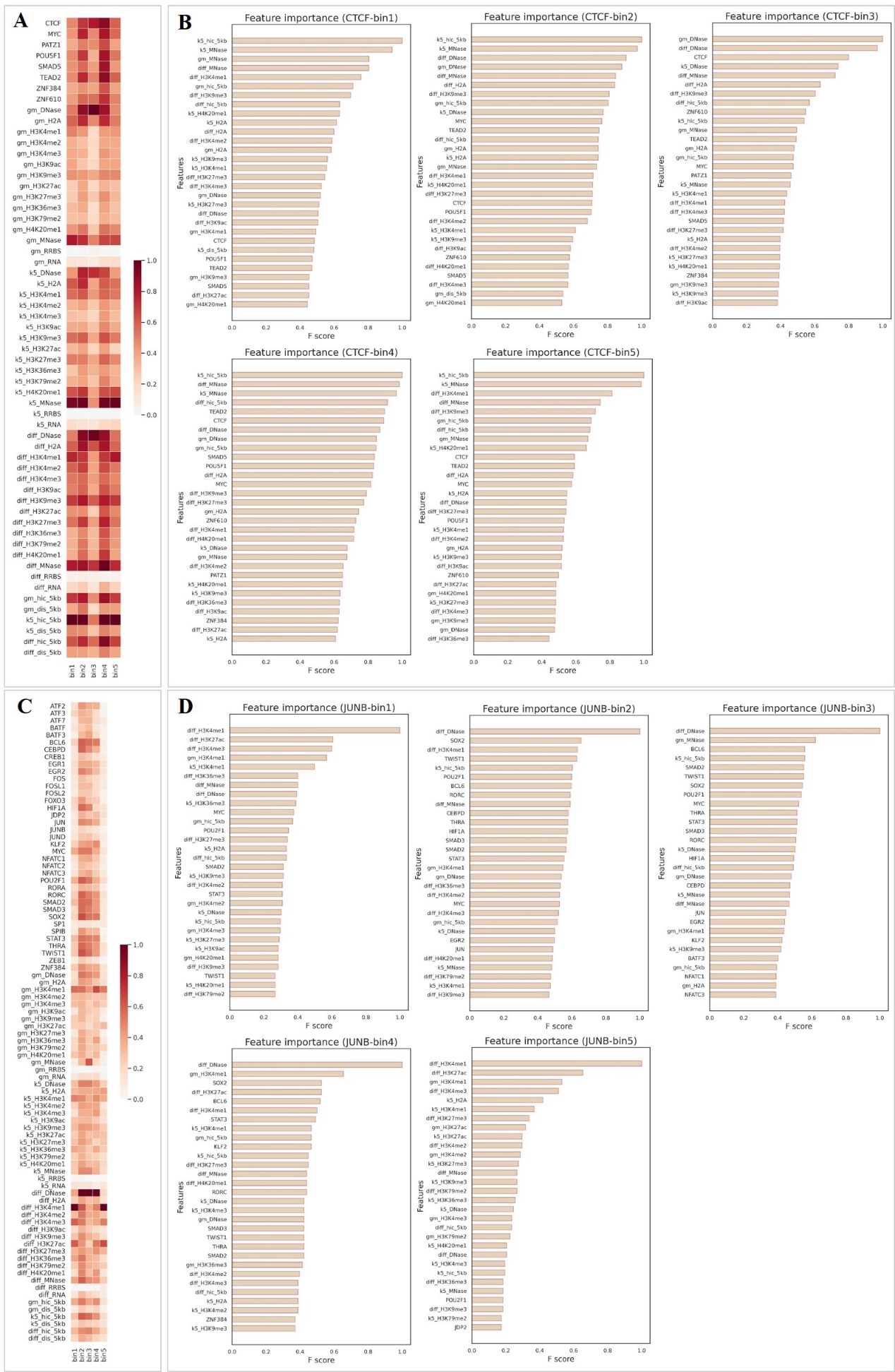


**Supplementary figure 12.** The important features of different bins whose importance is computed using the corresponding bin. (A) The heatmap of the feature importance of the central bin (bin3) and the flanking bins (bin1, bin2, bin4, bin5) for CTCF. (B) The top-30 important features of the central bin and the flanking bins for CTCF. (C) The heatmap of the feature importance of the central bin (bin3) and the flanking bins (bin1, bin2, bin4, bin5) for JUNB. (D) The top-30 important features of the central bin and the flanking bins for JUNB.

**Supplementary Tables**

**Supplementary table 1.** The accession list of ChIP-seq datasets for 10 binding factors from the GM12878 and K562 cell lines. For peaks not provided by ENCODE, we will use the peak caller SPP to generate corresponding peaks.

| GM12878 | Accessions for Peaks | Accessions for Bams |
| --- | --- | --- |
| CEBPB | None | ENCFF573GQJ, ENCFF707LIB |
| CTCF | ENCFF710VEH | ENCFF119SGJ, ENCFF584BRF |
| FOS | ENCFF002COM | ENCFF000VSZ, ENCFF000VTA |
| JUNB | ENCFF939TZS | ENCFF273TMW, ENCFF415ELX |
| MAX | ENCFF083KVY | ENCFF386FSS, ENCFF892REX |
| MYC | ENCFF001USG | ENCFF000ROE, ENCFF000ROF |
| POLR2A | ENCFF120VUT | ENCFF865BUP, ENCFF591BQK |
| RAD21 | None | ENCFF311CJK, ENCFF800DLO |
| SP1 | None | ENCFF000OEL, ENCFF000OEO |
| YY1 | ENCFF967ACD | ENCFF180NKF, ENCFF004ZLO |
| K562 | Accessions for Peaks | Accessions for Bams |
| CEBPB | None | ENCFF059XPJ, ENCFF353LOM |
| CTCF | ENCFF738TKN | ENCFF487UYG, ENCFF496SZR |
| FOS | ENCFF002CVW | ENCFF000YIH, ENCFF000YIG |
| JUNB | ENCFF426DUB | ENCFF095BVD, ENCFF709WYV |
| MAX | ENCFF799HIG | ENCFF938QJA, ENCFF543CKI |
| MYC | ENCFF465JKF | ENCFF058VAU, ENCFF384WMI |
| POLR2A | ENCFF947KPB | ENCFF822HFC, ENCFF359UUS |
| RAD21 | None | ENCFF084HTD, ENCFF330BAK |
| SP1 | None | ENCFF593LCA, ENCFF515QZM |
| YY1 | ENCFF328XKC | ENCFF189XLI, ENCFF872BSD |

**Supplementary table 2.** The accession list of chromatin landscapes from the GM12878 and K562 cell lines.

| GM12878 | URL |
| --- | --- |
| DNase | https://egg2.wustl.edu/roadmap/data/byFileType/signal/consolidated/macs2signal/pval/E116-DNase.pval.signal.bigwig |
| H2A.Z | https://egg2.wustl.edu/roadmap/data/byFileType/signal/consolidated/macs2signal/pval/E116-H2A.Z.pval.signal.bigwig |
| H3K4me1 | https://egg2.wustl.edu/roadmap/data/byFileType/signal/consolidated/macs2signal/pval/E116-H3K4me1.pval.signal.bigwig |
| H3K4me2 | https://egg2.wustl.edu/roadmap/data/byFileType/signal/consolidated/macs2signal/pval/E116-H3K4me2.pval.signal.bigwig |
| H3K4me3 | https://egg2.wustl.edu/roadmap/data/byFileType/signal/consolidated/macs2signal/pval/E116-H3K4me3.pval.signal.bigwig |
| H3K9ac | https://egg2.wustl.edu/roadmap/data/byFileType/signal/consolidated/macs2signal/pval/E116-H3K9ac.pval.signal.bigwig |
| H3K9me3 | https://egg2.wustl.edu/roadmap/data/byFileType/signal/consolidated/macs2signal/pval/E116-H3K9me3.pval.signal.bigwig |
| H3K27ac | https://egg2.wustl.edu/roadmap/data/byFileType/signal/consolidated/macs2signal/pval/E116-H3K27ac.pval.signal.bigwig |
| H3K27me3 | https://egg2.wustl.edu/roadmap/data/byFileType/signal/consolidated/macs2signal/pval/E116-H3K27me3.pval.signal.bigwig |
| H3K36me3 | https://egg2.wustl.edu/roadmap/data/byFileType/signal/consolidated/macs2signal/pval/E116-H3K36me3.pval.signal.bigwig |
| H3K79me2 | https://egg2.wustl.edu/roadmap/data/byFileType/signal/consolidated/macs2signal/pval/E116-H3K79me2.pval.signal.bigwig |
| H4K20me1 | https://egg2.wustl.edu/roadmap/data/byFileType/signal/consolidated/macs2signal/pval/E116-H4K20me1.pval.signal.bigwig |
| RRBS | https://egg2.wustl.edu/roadmap/data/byDataType/dnamethylation/RRBS/FractionalMethylation_bigwig/E116_RRBS_FractionalMethylation.bigwig |
| MNase | ENCFF000VME |
| RNA | ENCFF755RBT, ENCFF319UTC, ENCFF975RWM, ENCFF282OWV |
| K562 | URL |
| DNase | https://egg2.wustl.edu/roadmap/data/byFileType/signal/consolidated/macs2signal/pval/E123-DNase.pval.signal.bigwig |
| H2A.Z | https://egg2.wustl.edu/roadmap/data/byFileType/signal/consolidated/macs2signal/pval/E123-H2A.Z.pval.signal.bigwig |
| H3K4me1 | https://egg2.wustl.edu/roadmap/data/byFileType/signal/consolidated/macs2signal/pval/E123-H3K4me1.pval.signal.bigwig |
| H3K4me2 | https://egg2.wustl.edu/roadmap/data/byFileType/signal/consolidated/macs2signal/pval/E123-H3K4me2.pval.signal.bigwig |
| H3K4me3 | https://egg2.wustl.edu/roadmap/data/byFileType/signal/consolidated/macs2signal/pval/E123-H3K4me3.pval.signal.bigwig |
| H3K9ac | https://egg2.wustl.edu/roadmap/data/byFileType/signal/consolidated/macs2signal/pval/E123-H3K9ac.pval.signal.bigwig |
| H3K9me3 | https://egg2.wustl.edu/roadmap/data/byFileType/signal/consolidated/macs2signal/pval/E123-H3K9me3.pval.signal.bigwig |
| H3K27ac | https://egg2.wustl.edu/roadmap/data/byFileType/signal/consolidated/macs2signal/pval/E123-H3K27ac.pval.signal.bigwig |
| H3K27me3 | https://egg2.wustl.edu/roadmap/data/byFileType/signal/consolidated/macs2signal/pval/E123-H3K27me3.pval.signal.bigwig |
| H3K36me3 | https://egg2.wustl.edu/roadmap/data/byFileType/signal/consolidated/macs2signal/pval/E123-H3K36me3.pval.signal.bigwig |
| H3K79me2 | https://egg2.wustl.edu/roadmap/data/byFileType/signal/consolidated/macs2signal/pval/E123-H3K79me2.pval.signal.bigwig |
| H4K20me1 | https://egg2.wustl.edu/roadmap/data/byFileType/signal/consolidated/macs2signal/pval/E123-H4K20me1.pval.signal.bigwig |
| RRBS | https://egg2.wustl.edu/roadmap/data/byDataType/dnamethylation/RRBS/FractionalMethylation_bigwig/E123_RRBS_FractionalMethylation.bigwig |
| MNase | ENCFF000VNN |
| RNA | ENCFF464HGS, ENCFF295KNU, ENCFF617ZPZ, ENCFF061KDL |

**Supplementary table 3.** The number of GM12878-specific, K562-specific, and shared binding peaks across all datasets.

| Binding Factor | GM12878-specific Peaks | K562-specific Peaks | Shared Peaks |
| --- | --- | --- | --- |
| CEBPB | 1109 | 1834 | 11064 |
| CTCF | 3665 | 5693 | 30694 |
| FOS | 143 | 712 | 1680 |
| JUNB | 3847 | 5557 | 10512 |
| MAX | 4684 | 5457 | 16875 |
| MYC | 9439 | 2492 | 16294 |
| POLR2A | 488 | 1556 | 1044 |
| RAD21 | 7474 | 7408 | 15780 |
| SP1 | 2608 | 2677 | 7535 |
| YY1 | 4304 | 5524 | 21568 |
